# *Pelagibaca bermudensis* promotes biofuel competence of *Tetraselmis striata* in a broad range of abiotic stressors: dynamics of quorum-sensing precursors and strategic improvement in lipid productivity

**DOI:** 10.1186/s13068-018-1097-9

**Published:** 2018-04-07

**Authors:** Shailesh Kumar Patidar, Sae-Hee Kim, Jin Ho Kim, Jungsoo Park, Bum Soo Park, Myung-Soo Han

**Affiliations:** 1 Department of Life Science, College of Natural Sciences, Hanyang University, Seoul, South Korea; 2 Research Institute of Natural Sciences, Hanyang University, Seoul, South Korea; 3 Present Address: Marine Science Institute, University of Texas at Austin, Port Aransas, TX USA

**Keywords:** Co-cultivation, Biofuel, Biomass, Lipid, Two-stage cultivation, Quorum-sensing precursor

## Abstract

**Background:**

Amelioration of biofuel feedstock of microalgae using sustainable means through synthetic ecology is a promising strategy. The co-cultivation model (*Tetraselmis striata* and *Pelagibaca bermudensis*) was evaluated for the robust biofuel production under varying stressors as well as with the selected two-stage cultivation modes. In addition, the role of metabolic exudates including the quorum-sensing precursors was assessed.

**Results:**

The co-cultivation model innovated in this study supported the biomass production of *T. striata* in a saline/marine medium at a broad range of pH, salinity, and temperature/light conditions, as well as nutrient limitation with a growth promotion of 1.2–3.6-fold. Hence, this developed model could contribute to abiotic stress mitigation of *T. striata*. The quorum-sensing precursor dynamics of the growth promoting bacteria *P. bermudensis* exhibited unique pattern under varying stressors as revealed through targeted metabolomics (using liquid chromatography–mass spectrometry, LC–MS). *P. bermudensis* and its metabolic exudates mutually promoted the growth of *T. striata*, which elevated the lipid productivity. Interestingly, hydroxy alkyl quinolones independently showed growth inhibition of *T. striata* on elevated concentration. Among two-stage cultivation modes (low pH, elevated salinity, and nitrate limitation), specifically, nitrate limitation induced a 1.5 times higher lipid content (30–31%) than control in both axenic and co-cultivated conditions.

**Conclusion:**

*Pelagibaca bermudensis* is established as a potential growth promoting native phycospheric bacteria for robust biomass generation of *T. striata* in varying environment, and two-stage cultivation using nitrate limitation strategically maximized the biofuel precursors for both axenic and co-cultivation conditions (T and T-PB, respectively). Optimum metabolic exudate of *P. bermudensis* which act as a growth substrate to *T. striata* surpasses the antagonistic effect of excessive hydroxy alkyl quinolones [HHQ, 4-hydroxy-2-alkylquinolines and PQS (pseudomonas quorum signal), 2-heptyl-3-hydroxy-4(1H)-quinolone].

**Electronic supplementary material:**

The online version of this article (10.1186/s13068-018-1097-9) contains supplementary material, which is available to authorized users.

## Background

Microalgae are an emerging potential source of sustainable biofuel in addition to efficient carbon sequestration and bioremediation [1–4]. Innovative marine microalgal cultivation has been suggested for biofuel production because of its extremely low freshwater and cultivable land crop footprint [5]. The cost-effective cultivation and determination of optimum environmental conditions for achieving the desired improvement in biomass and biofuel precursors is an essential step for the technological advancement [6, 7]. Most of the research in microalgal technologies has focused on elevation and amelioration of desired biological yields of the axenic or monoalgal strains without considering the potential of phycospheric bacteria [3, 4, 8, 9].

However, limited research has been conducted on growth promoting sustainable cultivation techniques to ascertain the biomass productivity through biotic interactions despite the enormous potential of synthetic/natural co-cultivation. The metabolite and nutrients exuded by the phycospheric growth promoting bacteria could offer impressive biomass yields with suitable fatty acid properties to make biodiesel [10–12]. Furthermore, the mutualistic phycospheric bacteria can grow in similar physicochemical environments whenever co-cultivated, while it could not grow alone in the same media, and remarkable growth co-operation for the nutrients was shown which could reduce the cost of the cultivation [12].

Most of the co-cultured mutual consortia may release volatile or non-volatile growth promoting metabolites (such as vitamins, hormones, amino acids, and fatty acids), macronutrients (K, N, and P), and micronutrients such as trace metals (including B, Mn, Ni, Cu, Fe, and Co), and these metabolites (including infochemicals) support to the respective partner during the growth [13–17]. Some bacteria exhibit potential for environmental applications in wastewater remediation and biofuel production and a consortium (natural or synthetically engineered) could promote the growth of desired microalgae as well as exhibit algicidal effects on undesired microalgae [12, 14, 16, 17].

Some of the different microalgal seawater strains are accorded a relatively higher significance for open/semi-enclosed cultivation in coastal waters. *Tetraselmis* is well known for its commercial potential for biomass and biofuel production in coastal areas [18, 19]. *Tetraselmis* spp. are highly influenced by the associated bacteria; there is an opportunity to boost the biomass productivity via synthetic phycosphere engineering upon establishing the mechanism and optimal processes [12, 20–22]. The previous study on *Tetraselmis striata* and its native phycospheric bacterium *Pelagibaca bermudensis* under axenic and co-cultivation conditions (T and T-PB, respectively) elucidated the mutual co-operative effects of the phosphate and organic/inorganic carbons. However, the study was conducted under sole unvarying light, temperature, and salinity conditions [12]. There is a growing consensus on the importance of understanding the growth promoting effects caused by bacteria under various environmental stressors. Furthermore, determining whether *P. bermudensis* has growth promoting effects on microalgae under varying abiotic conditions is imperative, because the coastal aquatic environment and effluent load are extremely variable where it needs to be applicable.

*Pelagibaca bermudensis* belongs to the well-known *Roseobacter* clade of the Alphaproteobacteria family, which is one of the predominant seawater bacterial communities and is also found in a wide range of habitats [23, 24]. Some members of this family have known quorum-sensing abilities for biofilm formation on biotic/abiotic surfaces and can use a variety of organic carbon sources, which they degrade effectively [25–27]. Roseobacter *P. bermudensis* may degrade aliphatic and aromatic hydrocarbons due to its hydrocarbonoclastic properties [28]. *P. bermudensis* HTCC2601 isolated from surface water has genes that encode all C3 cycle enzymes [29]. It is also established as a d-carbamoylase-producing bacterium and synthesizes antimicrobial agents such as tropodithietic acid [23, 30]. The marine bacterium *P. bermudensis* encodes an uncharacterized member of the phosphopyruvate hydratase (enolase) superfamily [31]. It predominantly has fatty acid 18 : 1ω7c (79.7 % of total fatty acids) [24].

Quorum sensing is the ability of the organisms to regulate gene expression of the varied function using the precursors and to act as a multicellular system for collective decision making which is often correlated to their cell populations [27, 32]. Most members of the *Roseobacter* clade also contain the RuBISCO enzyme, which is involved in CO_2_ fixation and has an oligophototrophic/chemoheterotrophic/photoheterotrophic nature [33, 34].

The growth dynamics of *P. bermudensis* in the presence of *T. striata* as a mutual partner, as well as the extent of this mutual partnership and role of quorum-sensing precursors (acyl homoserine lactones and alkyl quinolones) concentrations in this consortium are currently unexplored. The role of acyl homoserine lactones and alkyl quinolones in the intrapopulation and interpopulation communication during co-cultivation may be a significant factor for the possible relationships, colonization, quorum sensing, biofilm formation, cellular recognition, and cell density-dependent regulation of target genes as well as in growth dynamics [32, 35, 36]. The primary objective of  this research was to study the mutual growth, biomass, and lipid productivity of the *T. striata* under co-cultivated (T-PB) and axenic (T) conditions in varying environmental stressors; second, to monitor the quorum-sensing precursors of *P. bermudensis* during co-cultivation, and their effect on growth of the *T. striata*; furthermore, to assess the amelioration of the biomass and lipid of the *T. striata* for feasible biofuel production under varying crude exo-metabolic feeding and with selected two-stage cultivation modes.

## Methods

### Culture development and maintenance

#### *P. bermudensis*

The *P. bermudensis* (KCTC13073BP) used in study was isolated from the phycosphere of *T. striata*. *P. bermudensis* maintained and cultured in the axenic form in cell culture flasks (containing marine broth) and marine nutrient agar plates. Periodical maintenance and axenic culture monitoring was performed. The details of the isolation of *P. bermudensis* from phycosphere of *Tetraselmis*, axenic (T) culture preparation, and molecular identification of the strain used in this study are available elsewhere [12].

#### *T. striata*

*Tetraselmis striata* was isolated from seawater samples from the Incheon coast mass cultivation site of Inha University. Culture of *T. striata* was deposited at the Korean Culture Repository after molecular identification by Inha Univeristy and was assigned Accession No. KCTC1432BP. The microalgal culture was also maintained in O3 medium in the algal culture laboratory of the Department of Life Science, Hanyang University. The O3 media was containing: 20 g L^−1^ of NaCI, 5.0 g L^−1^ of MgSO_4_·7H_2_O, 4.25 g L^−1^ of MgCl_2_·6H_2_O, 1.13 g L^−1^ of CaCl_2_·2H_2_O, 0.76 g L^−1^ of KNO_3_, 0.05 g L^−1^ of KH_2_PO_4_, 0.03 g L^−1^ of NaHCO_3_, and 0.76 mL of stock solution A (18.6 g L^−1^ of Na_2_EDTA and 2.4 g L^−1^ of FeCl_3_·6H_2_O L^−1^ dissolved in distilled H_2_O) and 0.76 mL of stock solution B [40 mg L^−1^ of ZnCl_2_, 600 mg L^−1^ of H_3_BO_3_, 15 mg L^−1^ of CoCl_2_·6H_2_O, 40 mg L^−1^ of CuCl_2_·2H_2_O, 488 mg L^−1^ of MgCl_2_·4H_2_O, and 37 mg L^−1^ of (NH_4_)_6_MoO_24_·4H_2_O dissolved in distilled H_2_O and 0.38 mL of vitamin B_12_ stock solution (0.1 mg of vitamin B_12_ in 100 mL of distilled water). 1 M Tris–HCl and 1 M NaOH were used for adjusting the pH [37]. The modification of the media according to the experiments (salinity, pH, and nutrient limitation) is mentioned in the separate section.

The axenic *T. striata* culture cells (T) were maintained in cell culture flasks containing O3 media (pH 8.0) incubated at 20 °C with exposure of 50 µM m^−2^ s^−1^ by cool fluorescent lamps (12:12 h photoperiod). Axenic culture and experimental culture were routinely maintained by repetitive agar plating techniques and microscopic observations.

Furthermore, prior to initiate the experiment, axenic status of the culture was also monitored using 4′,6-diamidino-2-phenylindole (DAPI, Sigma-Aldrich, St. Louis, MO, USA) staining for bacterial presence under a microscope (BX51 Olympus, Japan). The inoculum volume to attain the final cell abundance of *T. striata* and *P. bermudensis* in experimental culture flask on the initial hours of the experiment was determined by actual cell abundance of mother culture. Cell abundance was measured according to the “Cell abundance of microalgae and bacteria, and growth promotion” section. The cells of the determined inoculum volume were harvested using the centrifugation; complying with standard sterile conditions and resulting axenic washed pellet were re-suspended in axenic and co-cultivated conditions.

### T-PB and T cultivation experiments under varying stress conditions

To investigate the effect of pH, the O3 media were initially adjusted for the different pH of 4, 6, 8, and 10 followed by the autoclave. After centrifuging the *T. striata* and *P. bermudensis* culture and repeatedly washing the pellet, the starved cells were inoculated in 100-mL O3 medium culture flask (150 mL) under sterile laminar flow conditions. *T. striata* and *P. bermudensis* were co-inoculated and adjusted to the initial cell densities up to 10^4^ and 1.72 × 10^8^ cells mL^−1^, respectively, for each experiment of co-cultivation (T-PB). The control culture under T conditions was only inoculated with axenic *T. striata*. All the conditions were investigated in triplicates.

Similarly, the O3 media containing different salinity with sodium chloride (NaCl) at 20, 25, 30, and 35 gL^−1^ and an initial pH of 8.0 were used for both the T and T-PB experiments. To examine the effect of varying light and temperature on *T. striata* under T-PB and T conditions, low-light (30 µM m^−2^ s^−1^ with 20 °C, 12:12 h), and low-temperature (15 °C with 45 µM m^−2^ s^−1^, 12:12 h) conditions were compared with comparatively higher light (147 µM m^−2^ s^−1^, 20 °C, 24:00 and 12:12 h.) and normal light and temperature (45 µM m^−2^ s^−1^, 20 °C, 12:12 h) conditions (Table 1). All the experiments related to light/temp were run in triplicates using the O3 media at an initial pH of 8.0 and salinity of 20 g L^−1^.

**Table 1:** Experimental codes for the varying pH, salinity, light, and temperature conditions employed for T and T-PB

Factor	Experimental code used in this study	Media	pH	Salinity (gL^−1^ NaCl)	Light (µM cm^−2^ S^−1^)	Light duration (light:dark)	Temperature (°C)
pH	*8*	*O3*	*8*	*20*	*45*	*12:12*	*20*
	4	O3	4	20	45	12:12	20
	6	O3	6	20	45	12:12	20
	10	O3	10	20	45	12:12	20
Salinity	*20*	*O3*	*8*	*20*	*45*	*12:12*	*20*
	25	O3	8	25	45	12:12	20
	30	O3	8	30	45	12:12	20
	35	O3	8	35	45	12:12	20
Light and temp.	*NLT or normal light and temp.*	*O3*	*8*	*20*	*45*	*12:12*	*20*
	LL or low light	O3	8	20	30	12:12	20
	LT or low temp.	O3	8	20	45	12:12	15
	CHL or continuous light and temp.	O3	8	20	145	24:00	20
	HL or high light	O3	8	20	145	12:12	20

Conditions as italicized are respective controls with the same culture environment

### Cell abundance of microalgae and bacteria, and growth promotion

To determine the cell abundance (cells mL^−1^), the cells were counted every third day. Samples (100 µL) from each experimental flask were ejected out under sterile laminar flow conditions for the *T.* striata and *P. bermudensis*, and immediately fixed with Lugol’s solution (final concentration 1%) and glutaraldehyde (2.5% glutaraldehyde), respectively. The microalgae growth was determined by enumerating the cells (fixed with 1% Lugol’s solution) observed using a hemocytometer under a light microscope at 200× magnification (BX51 Olympus, Japan). Similarly, the bacterial samples (fixed with glutaraldehyde) were counted using the DAPI epifluorescence technique. Before microscopy and enumeration of bacteria, each sample was filtered through a 0.2-μm GTTP Millipore filter membrane (Millipore Filter Corporation, Ireland) under 178 mmHg and then stained with 4′,6-diamidino-2-phenylindole (DAPI) for 8–12 min under dark conditions. The filter membrane containing fixed and stained bacteria was taken on the glass slide and covered by cover slip containing Fluor-mount (Sigma, USA). The slides were kept in the cool and dark place until analysis. During the observation under microscope, UV transparent immersion oil was used. The cell abundance of *P. bermudensis* was then enumerated on the basis of cell counts at 1000× under an epifluorescence microscope (Olympus, Japan equipped with wavelength filter set) from a microscope slide (Marienfeld, Germany) as per Eq. 1. The cell abundance was analyzed in ten randomly selected fields of view across each of the slides based on the method of Joo et al. [38]:

1 $${\text{Abundance cell number}} = \frac{{\left( {\text{filtering area}} \right)\left( {\text{total cell count}} \right)}}{{\left( {\text{total observed area}} \right)\left( {\text{filtering volume}} \right)}}.$$

The growth promotion (fold) was determined as the ratio of the cell abundance of co-cultivated *T. striata* (T-PB) and axenic *T. striata* (T) in the stationary phase of the same cultivation time.

### Biomass productivity and lipid content measurement

A known amount of the experimental culture was harvested, while the *T. striata* cells reached to stationary phase (day 18). The cultured cells were harvested by centrifugation (3700×*g* for 10 min), the cell pellet was washed twice with distilled water (DW) at the same centrifugal force, and then, the wet biomass was dried at 70 °C in a drying oven for 24 h. The biomass productivity of both the axenic *T. striata* (T) and co-cultivated *T. striata* and *P. bermudensis* (T-PB) was determined based on the cell dry weight using a gravimetric method [8] by the following equation:

2 $${\text{Biomass productivity}}\;({\text{mg}}\;{\text{L}}^{ - 1} \;{\text{day}}^{ - 1} ) \, = {\text{cell dry weight}}\;({\text{mg}}\;{\text{L}}^{ - 1} )/{\text{growth interval }}\left( {\text{day}} \right).$$

The total lipids were extracted from a known weight of dried biomass using a solvent mixture of chloroform and methanol (2:1, v/v) according to the Folch method [39] and were finally determined using the following equation:

3 $${\text{Total lipid}}\left( \% \right) = {{{\text{Gravimetricweight of lipid }}\left( {\text{g}} \right)} \mathord{\left/ {\vphantom {{{\text{Gravimetricweight of lipid }}\left( {\text{g}} \right)} {{\text{CDW}}\left( {\text{g}} \right)}}} \right. \kern-0pt} {{\text{CDW}}\left( {\text{g}} \right)}} \times 100,$$

4 $${\text{Lipid productivity}}\;({\text{mg}}\;{\text{L}}^{ - 1} \;{\text{day}}^{ - 1} ) = {\text{Biomass productivity }}({\text{mg}}\;{\text{L}}^{ - 1} \;{\text{day}}^{ - 1} ) \times {\text{total lipid }}\left( \% \right).$$

The total chlorophyll concentration error in folch extracted lipid fraction was 0.031–0.042% of dry weight of biomass, which was less than the error of the total lipid (%) found in the experimental triplicates.

### Determination of Acyl homoserine lactones and alkyl quinolones concentration in cultures

The cultures grown for different days under the varying physicochemical conditions described in “T-PB and T cultivation experiments under varying stress conditions” section were harvested by centrifugation at 3700×*g* for 10 min; the supernatant was frozen immediately and then transferred to a − 80 °C deep freezer till the analysis. The supernatants were thawed and filtered through a 0.22-µm sterile Whatman filter paper.

An equal amount of sodium acetate was used to extract the metabolites. After ensuring maximum metabolite extraction by vortex and mixing, the extracted layers were separated and then freeze-dried under vacuum conditions. The dried extracts were treated with acidic methanol for derivatization. Acyl homoserine lactone (AHL) and alkyl quinolone (AQ) standards, namely *N*-(3-oxo decanoyl)-l-homoserine lactone, *N*-[-(RS)-3-hydroxybutyryl] l-homoserine lactone, *N*-butyryl-dl-homoserine lactone, 4-hydroxy-2-heptylquinoline (HHQ), and 2 heptyl-3 hydroxy-4 (1H) quinolone (PQS) (Sigma-Aldrich), were used as the standards. The derivatized products from the samples were analyzed using an ultra-performance-liquid chromatography (UPLC) system (Ultimate 3000 RS UHPLC, Thermo Fisher Scientific) coupled with mass spectrometry (MS, Quantiva, Thermo Fisher Scientific, USA).

The analysis was carried out in the positive polarity mode using an electrospray ionization (ESI) source (3500 V). The ion transfer tube and vaporizer temperatures were 342 and 350 °C, respectively. The analytes were separated using a Thermo Hypersil gold C18 column (2.1 × 100 mm, 1.9 µm) at 35 °C with a 0.4 mL/min flow rate. The mobile phase consisted of 0.1% formic acid in water (A) and 0.1% formic acid in acetonitrile (B). The gradient used was 10% B in A for 1.0 min, 50% B in A for 1.5 min, up to 99% B in A over 5.5 min and 7.5 min, and finally, the process was held at 90% B in A for 3 min. The standards of the AHL (acyl homoserine lactones) and AHQ (alkyl hydroxy quinolones) were run at different serially diluted concentrations, and a standard curve was constructed. Injection volume for each sample was 2 µL. Blanks were also similarly run for the quality control. The peaks were analyzed using relevant software and the concentrations were determined for the respective analyte molecules [40]

### Effects of crude metabolic exudates, HHQ, and PQS on microalgal cells

The *P. bermudensis* cultures were harvested after 2.5 days of the growth incubation in the marine broth media. The control (marine broth) without any bacterial inoculation was also incubated for the same time. The supernatants of the *P. bermudensis* culture (t) and media alone (MB) were collected, filtered through a 0.22-µm sterile syringe filter, and added to autoclaved samples of the O3 media in cell culture flasks under sterile conditions. The ratio of culture filtrates (MB with the *P. bermudensis* exudates) to the final experimental O3 media volume was 0:100 (0% t, control), 1:99 (1% t), 5:95 (5% t), 10:90 (10% t), and 100:00 (100% t). A similar ratio was used for the control (MB, the supernatant of the MB of the same incubation time) instead of using the *P. bermudensis* culture supernatant. The microalgal cells at the late log phase were inoculated to adjust the initial cell density to 10^4^ cells mL^−1^ in the cell culture flask on day 0 of the experiment. All the experiments were performed in triplicates (*n *=* 3*). Similarly, the cell abundance, biomass productivity, and total lipid content were assessed using the methods described in this section.

To investigate an independent effect of HHQ and PQS on *T. striata*, HHQ and PQS were dissolved in acetonitrile and added in different concentration in the O3 media, and initial cell density of the *T. striata* (10^4^ cells mL^−1^) was adjusted by inoculating the starved cells and growth was observed during the experiment.

### Investigation of nutrient limitation conditions

*T. striata* was grown under T and T-PB mode under limited nitrate, sulfate, and trace metal (iron and other trace metals) levels in the O3 medium and compared with the nutrient replete condition (O3 control). The nitrate limited conditions were devoid of nitrate in the O3 media, since KNO_3_ was not added. However, nitrogen in the form of ammonia was present as it was added as constituent of stock A of O3 media. The trace metal-limited conditions were devoid of Iron and stock B. The sulfate-limited media were containing ~ 4–5 µM of sulfate after washing the cell pellet with sterile distilled water. However, the sulfate in the media was not added as a component of O3 for the sulfate-limited media. The inoculation conditions and measurement of cell abundance, lipid content, and biomass productivity were carried out using similar methods to those of the other experiments.

### Comparative two-stage cultivation

The two-stage cultivation mode including nutrient replete and nutrient limiting were employed for both T and T-PB. The microalgal cultures containing *T. striata* (axenic, T) and *T. striata* with the *P. bermudensis* (co-cultivated, T-PB) were cultivated in the O3 medium for 15 days (*n* = 12) under NLT conditions, as shown in Table 1. All experimental cultures were harvested using centrifugation and the cell pellet was washed with distilled water (DW). The starved cell pellets were re-suspended in fresh O3 medium of three different conditions for the second-stage cultivation of 3 days. Herein, three-distinct two-stage cultivation performed for elevated salinity (containing modified O3 with 35 g L^−1^ NaCl; *n* = 3), nitrate limitation (modified O3 without nitrate; *n* = 3), and low pH (O3 media with pH 6.0; *n* = 3) along with a parallel control (20 g L^−1^ NaCl, pH 8.0 and nutrient replete condition) for both T and T-PB conditions. After 3 days, cultures were harvested for the biomass productivity and total lipid content assessment.

### Statistical analysis

All experiments were run in triplicates (except quorum-sensing precursors monitoring, *n* = 2), while the error bars are represented as mean ± SD. Data were analyzed using the one-way analysis of variance (ANOVA) followed by post hoc test to determine the significance of differences (LSD) at *p* ≤ 0.05 between the treatments using SPSS.

## Results and discussion

### Effect of varying pH

**Fig. 1:**
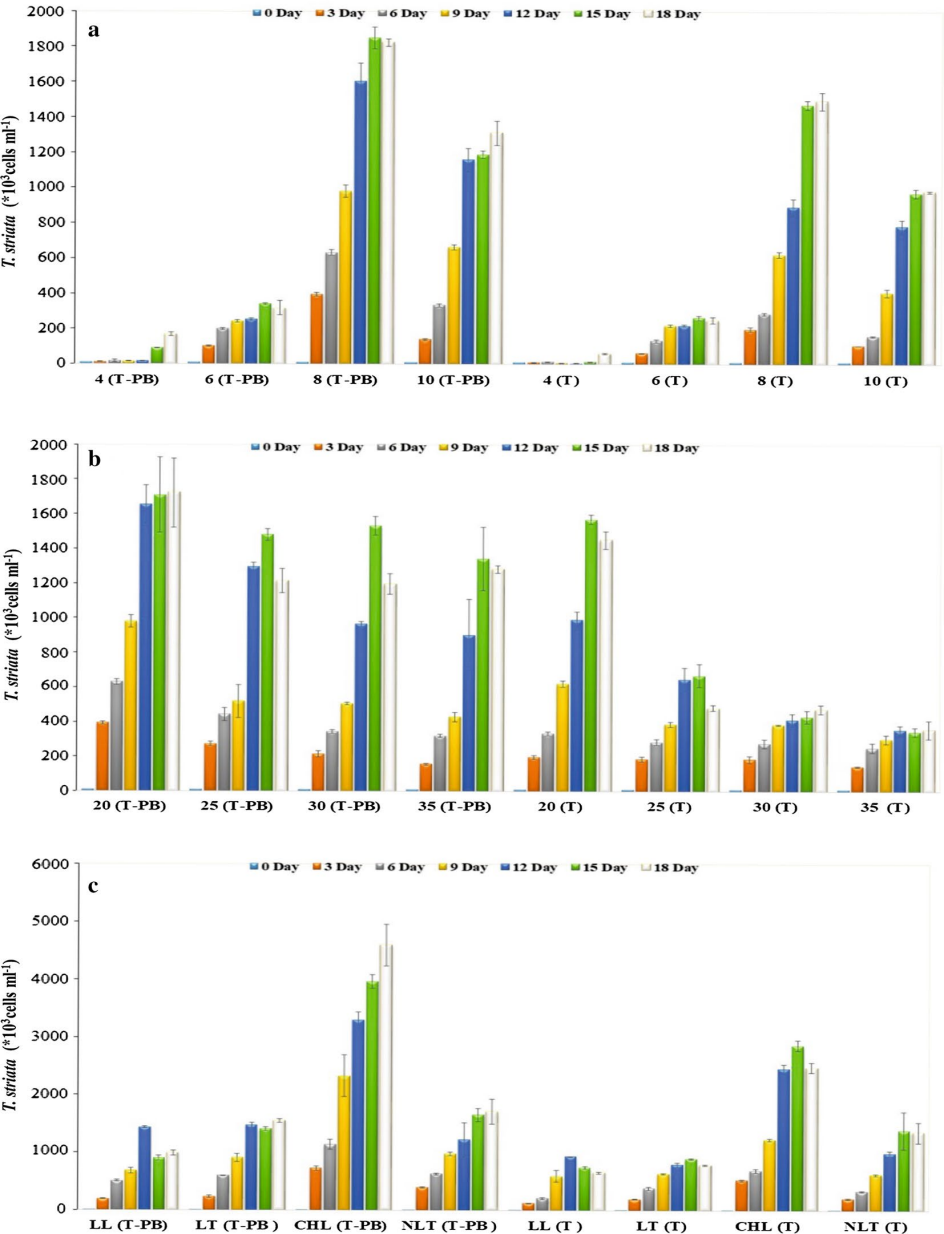
Cell abundance of *Tetraselmis striata* under axenic (T) and co-culture (T-PB) conditions at different (**a**) pH, **b** salinity (g L^−1^ NaCl), and **c** light/temperature in O3 medium. The values are presented in mean ± SD (*n* = 3). Error bars are showing SD. Details of the condition are exhibited in Table 1

**Fig. 2:**
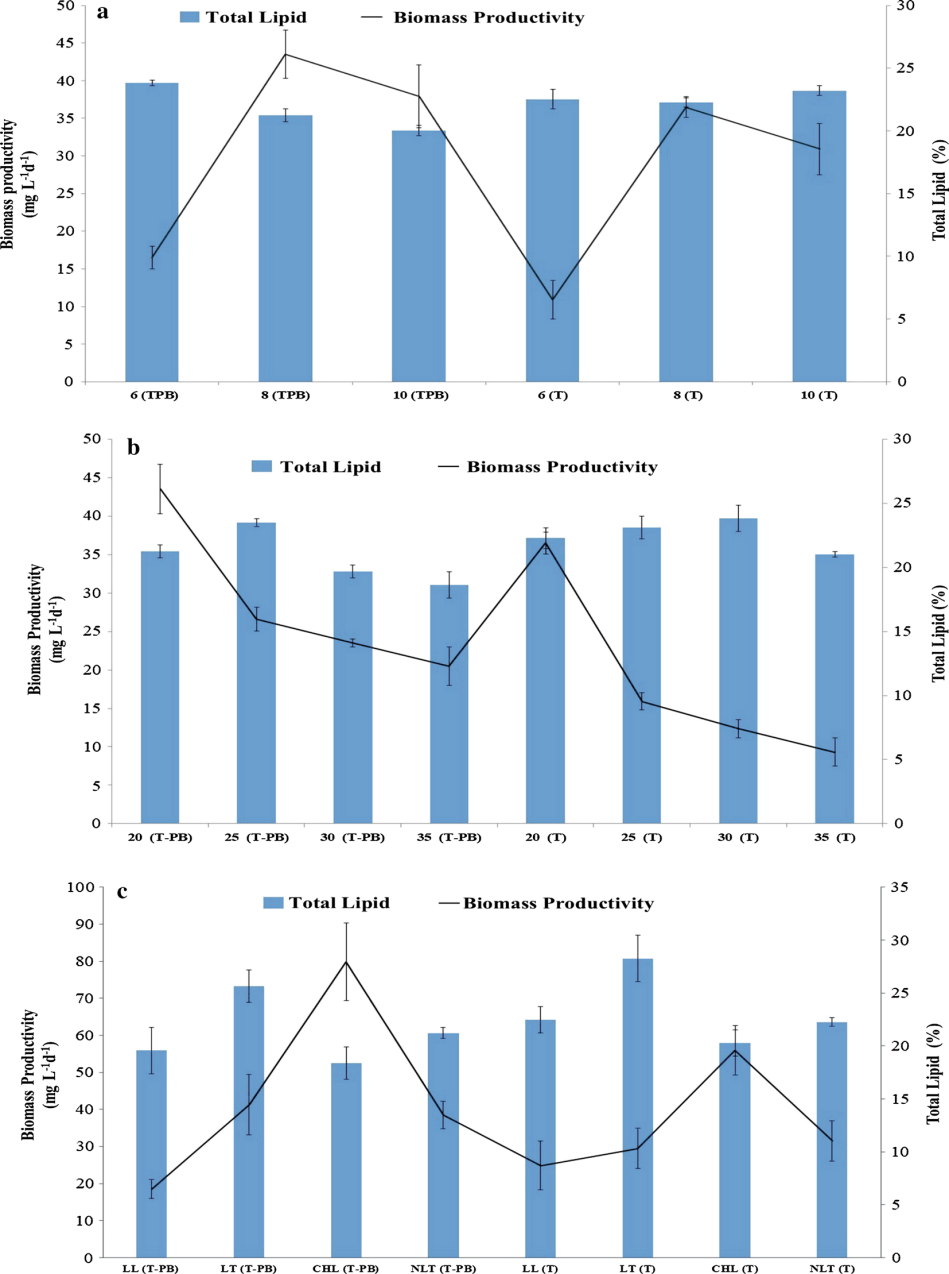
Biomass productivity and total lipid (%) of *Tetraselmis striata* under axenic (T) and co-cultivated (T-PB) conditions at different **a** pH, **b** salinity (g L^−1^ NaCl), and **c** light/temperature in O3 medium. The values are presented in mean ± SD (*n* = 3). Error bars are showing SD. Post hoc analysis is shown in the Additional file 2: Table S1 (A–F) for comparing the means according to their significant difference (LSD)

The total cell abundance of the microalgae under the T-PB condition with *P. bermudensis* and T conditions at different growth interval was the highest at pH 8.0 followed by pH 10.0, 6.0, and 4.0. *T. striata* showed a higher cell abundance in the presence of *P. bermudensis* than in its absence. The optimum pH conditions for the biomass productivity in both T and T-PB conditions were pH 8.0 and 10.0 (Figs. 1a and 2a). It was interesting to note that although the cell division rate of *T. striata* was restricted at pH 4.0 and 6.0, growth promotion was observed (Fig. 1a). This clearly indicates that the *P. bermudensis* provided a growth promoting environment for the *T. striata* under almost all the various pH conditions. The growth promotion rates (expressed as fold increases) on the cell harvesting day were 2.8-, 1.3-, 1.2-, and 1.3-fold at pH 4.0, 6.0, 8.0, and 10.0, respectively.

The maximum mean cell abundance on the harvesting day was obtained at the pH 8.0 and 10.0 (1.83 × 10^6^ and 1.31 × 10^6^ cells mL^−1^, respectively) under T-PB conditions (Fig. 1a). The cell density of *P. bermudensis* showed a sudden decrease at the initial T-PB phase but later improved under the optimal and suboptimal pH conditions (Additional file 1: Figure S1). Although both the bacterial and microalgal growth dynamics were affected by the H^+^ concentrations, the *P. bermudensis* remarkably survived under the acidic pH (4.0) and a multiple-fold enhancement of the growth in *T. striata* (6.41- and 2.81-fold on days 15 and 18, respectively; Fig. 1a and Additional file 1: Figure S1) was observed.

Furthermore, the inoculated cell density of *P. bermudensis* (10^8^ cells) was higher than that used previously (10^6^ cells mL^−1^) by Park et al. [12]. The higher cell density of *P. bermudensis* required higher levels of microalgal metabolites to support the growth in the initial phase and to initiate the mutualistic interactions. The lipid content of the biomass obtained under T conditions was approximately 1–3% higher than the that of the T-PB conditions at pH 8 and 10; however, the mean biomass productivity of T-PB increased by up to 19.30 to 22.67% at the same pH. The highest total lipid content was 23.8 ± 0.21% at pH 6.0, while the highest biomass productivity was 43.52 ± 3.2 mg L^−1^ day^−1^ at pH 8.0 under T-PB conditions (Fig. 2a). The overall results exhibited that the T-PB conditions induced higher lipid productivity than the T condition at each investigated pH.

### Effect of varying salinity

The maximum cell abundance of the *T. striata* was observed in 20 g L^−1^ NaCl on day 15/18 under T-PB conditions. There was a decline in the cell abundance at concentrations of 25, 30, and 35 g L^−1^ NaCl (Fig. 1b). The results suggest that the cells could cope with the wide range of salinity levels (20–35 g L^−1^). The elevated salinity levels in the T condition could lead to impeded microalgal cell abundance and lower growth than that observed under the respective T-PB conditions. The growth promotion was 1.18-, 2.51-, 2.53-, and 3.58-fold in 20, 25, 30, and 35 g L^−1^ NaCl, respectively, for the microalgae on day 18. *T. striata* (under T-PB) grew in 20 g L^−1^ NaCl with a higher growth promotion rate during the earlier lag phase (1.97- and 1.89-fold on days 3 and 6, respectively).

From day 6 onwards, *T. striata* (under T conditions) cultured with elevated salinity did not grow effectively compare to cells grown under T-PB (Fig. 1b). The presence of *P. bermudensis* in the cultivation medium of the *T. striata* not only enhanced the microalgae growth but also showed a pronounced multifold growth promotion. This clearly indicates that *P. bermudensis* likely exuded some specific metabolites or nutrients under higher salinity levels, which mitigated the intracellular stress levels of the microalgal cells. The biomass productivity of the microalgae (under T conditions) was 36.48 ± 1.39, 15.90 ± 1.12, 12.35 ± 1.20, and 9.31 ± 1.81 mg L^−1^ day^−1^ with lipid contents of 22.27 ± 0.38, 23.1 ± 0.80, 23.81 ± 1.0, 21 ± 0.7% at 20, 25, 30, and 35 g L^−1^ NaCl, respectively. While the biomass productivities of the T-PB were 43.52 ± 3.2, 26.57 ± 1.56, 23.47 ± 0.52, 20.47 ± 2.48 mg L^−1^ day^−1^ with the lipid contents of 21.23 ± 0.56, 23.48 ± 0.30, 19.67 ± 0.52, 18.63 ± 0.82% at 20, 25, 30, and 35 g L^−1^ NaCl, respectively (Fig. 2b).

These results suggest that the salinity endurance and wide salinity tolerance of *T. striata* (T-PB) enhance its ability to produce consistent biomass. Furthermore, this effect could only be due to the phycospheric native growth promoting bacteria, since *T. striata* alone (T) did not exhibit a consistent biomass productivity under the wide salinity range used in this study. The growth dynamics of *P. bermudensis* was also found to be unique in each case owing to the varied salinity (Additional file 1: Figure S2). In fact, the cells of *P. bermudensis* that are lysed may also provide metabolic nutrients to the microalgae. Many of these metabolites are osmolytes and phytohormones, which potentially regulate the membrane integrity of the microalgal cells as well as alter or mitigate the intracellular stress levels [41, 42].

### Effect of varying light and temperature

*Tetraselmis striata* did not exhibit better growth under low-light conditions than it did under high-light intensity in the presence or absence of *P. bermudensis*. The continuous exposure of *T. striata* to high-light conditions increased its growth by several degrees under both conditions compared to that under the other experimental settings. The growth promotion (1.85-fold) was also pronounced in the presence of *P. bermudensis* under high-light intensity with a maximum cell abundance (4.6 × 10^6^ cells mL^−1^). The light intensity and photoperiods were highly dominating factors to affect the *T. striata* and, growth promotion enhanced under high-light intensity as well as photoperiod (Fig. 1c and Additional file 1: Figure S3). *Tetraselmis* can grow at a relatively wide temperature range with a low rate of variation, so the effect of temperature on the cell abundance of *T. striata* were minimum [43]. Irrespective of the temperature/light conditions, T-PB conditions remained superior to T conditions with a 1.26–1.96-fold growth promotion in the microalgal cell abundance on day 18 (Fig. 1c and Additional file 1: Figure S3).

Unlike the oscillated bacterial growth dynamics observed under the varying temperature, salinity, and pH conditions, the *P. bermudensis* showed consistent growth when continuously exposed to high-light intensity. Furthermore, a high cell abundance (3.189 × 10^9^ cells mL^−1^ on day 18) was observed during the experimental period, which might have been due to luxuriant microalgal growth and continuous photosynthesis (Additional file 1: Figure S4). The continuous high-light exposure induced a maximum biomass productivity of 79.85 and 56.00 mg L^−1^ day^−1^ with 18.38 and 20.29% lipid content under T-PB and T conditions, respectively. In contrast, the values were the lowest under low-light conditions (24.89 and 18.51 mg L^−1^ day^−1^ with 19.57 and 22.46% total lipids under T-PB and T conditions, respectively) under varying light/temperature conditions. Low-temperature conditions induced maximum lipid contents of 25.67 and 28.26% under T-PB and T conditions, respectively (Fig. 2c).

### Dynamics of quorum-sensing precursors under T-PB condition

**Fig. 3:**
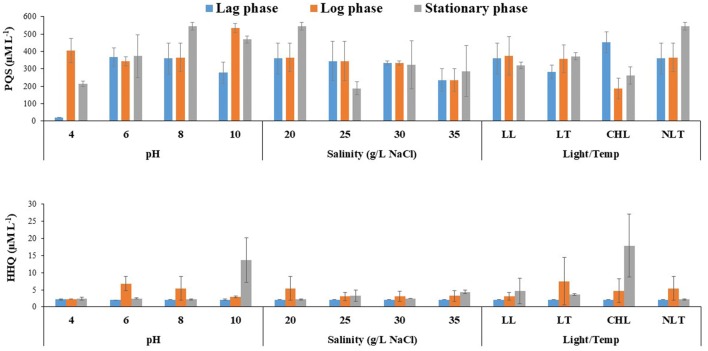
2-Heptyl-3-hydroxy-4(1H)-quinolone (PQS) and 2-heptyl-4-quinolone (HHQ) precursor concentrations under varying pH, salinity, light, and temperature in co-culture (T-PB) conditions at lag phase (day 3), log phase (day 9), and stationary phase (day 15). The values are presented in mean ± SD (*n* = 2). Error bars are showing SD. HHQ and PQS were below detectable range at 00 h of the experiment

The 2-heptyl-4-quinolone (HHQ) concentration in the lag phase was similar (2.02–2.14 µM) for all of the varying experimental T-PB conditions (Fig. 3). The accumulated HHQ was exuded under most conditions because of the decline in cell growth of *P. bermudensis* during the initial phase to the log phase except the high-light exposure (Fig. 3 and Additional file 1: Figure S4). The high growth rate of *T. striata* (high cell abundance) could have produced a high organic carbon content in the form of metabolites, which likely led to the growth of *P. bermudensis* and improved cell density in the T-PB condition. The HHQ was higher at the log or stationary phase of the microalgae growth cycle, especially under the optimum conditions of biomass and lipid, at a range of 2.28–17.19 µM (Fig. 3). The highest HHQ (17.19 µM) was observed with the continuous exposure to high-light intensities where the *P. bermudensis* constantly grow without any decline.

However, this observation was not correlated with the lag, log, and stationary phases of the *P. bermudensis* under different experimental conditions, since the cell abundance measured every third day, which is a long growth cycle for the *P. bermudensis*. This phenomenon could not be investigated with axenic *P. bermudensis* (without *T. striata*) in O3 media, since *P. bermudensis* alone did not grow without the *T. striata* metabolites (data not shown). However, there was an oscillation in the cell density of *P. bermudensis* (day 3–18, Additional file 1: Figure S4). The growth cycle of the *P. bermudensis* remained at a low range of the growth intervals.

Apart from the quorum-sensing function for the bacterial biofilm formation, HHQ is also known to exhibit algicidal activity. Previously, *Roseobacter* clade have been reported to show algicidal activity against *Emiliania huxleyi* strains with IC_50_ of 88–115 ng m L^−1^. In contrast, marine chlorophytes were not affected with elevated HHQ (IC_50_ = 10^5^ng mL^−1^) [44]. The HHQ and PQS concentration mediate the mutualistic interactions by indirect promotion of the algal growth through increasing cell abundance of *P. bermudensis* to form biofilm on the biotic surface and releases concentrated metabolic stock for their algal partner. However, while HHQ and PQS reached higher concentration, they can arrest the growth of *T. striata*. This example could be similar as a role of other algicidal metabolite roseobacticide. Although Roseobacter have symbiotic association for the phytoplanktons under mutualistic conditions, they switch to parasitic relationship by sensing the senescence signal (p-coumaric acid) of their host algae and releases roseobacticides to control the growth of algae [45].

In the current study, the release of higher HHQ concentrations in the log/stationary phase could not be detrimental for the *T. striata* and did not likely hamper the growth promoting effect on the algal cells under T-PB conditions (Fig. 3). In fact, the dead cells could be degraded by the HHQ and the degradation complex molecules were probably simpler growth metabolites that could be used by the active cells of *T. striata* and *P. bermudensis*. HHQ is also known to degrade dimethylsulfoniopropionate (DMSP) [44]. Therefore, the degradation of DMSP may provide sulfur to the *T. striata* and *P. bermudensis*. 2-Heptyl-3-hydroxy-4(1H) quinolone (PQS) was found at a high concentration (188–544 µM) except at the low pH conditions where the concentration was 20.84 µM.

Furthermore, the concentration trend varied under different environmental conditions (Fig. 3). PQS is known to entrap iron by chelation and indirectly promotes siderophores production during the limiting nutrient conditions. PQS is known to act through at least three-distinct signaling pathways and separately has an iron scavenging mechanism, while its precursor, HHQ, cannot form an iron complex. In the previous studies, the exposure of *P. aeruginosa* mutant to PQS prevented the cells from synthesizing pyoverdine or pyochelin. PQS was found to be involved in cell envelope formation and inhibited bacterial growth. The secondary functions/multi-functions of PQS include iron entrapment, which facilitates siderophore-mediated iron delivery [46]. Thus, based on the current results, the higher PQS concentration may be involved in the entrapment and release of iron under iron-starved conditions wherein *T. striata* could have benefited.

### Effect of nutrient limitation and replete conditions

*Tetraselmis striata* under T-PB conditions (with *P. bermudensis*) and nitrogen, sulfur, and trace metal limitations showed a higher growth rate than it did under T conditions. However, all the nutrient limitation conditions induced restricted growth compare to the nutrient replete conditions (Additional file 1: Figure S6). The trace metal and nitrate limitation exhibited higher growth inhibition. In comparison, the nitrate limitation results revealed as the initial increase in the cell abundance of the microalgae with a slower growth rate. This observation indicates that an organic nitrogen source was potentially provided by the partner bacteria (*P. bermudensis*) or was due to strategical utilization of the organic nitrogen released by the cells of the same population (Additional file 1: Figure S6).

**Fig. 4:**
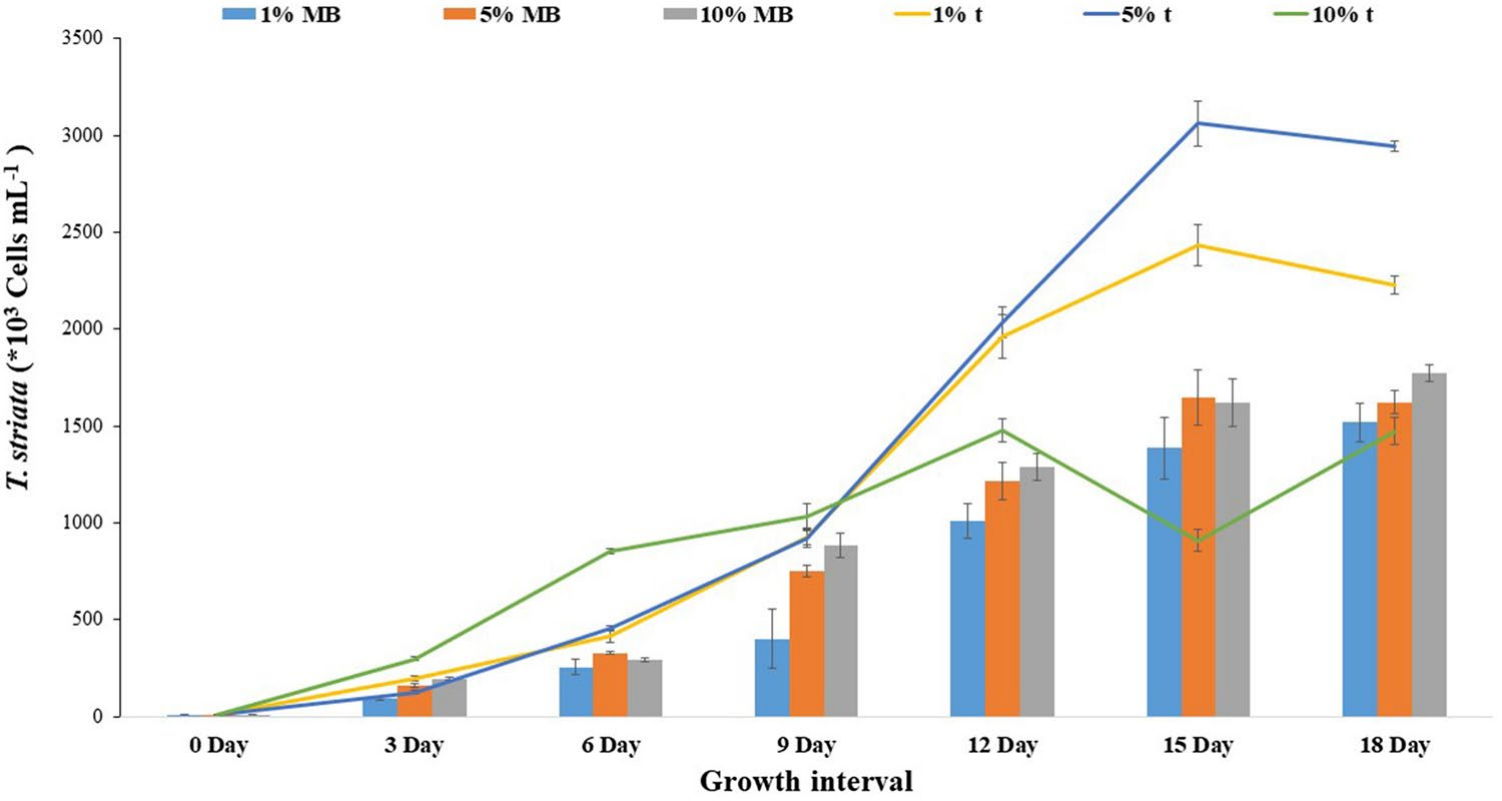
Biomass productivity and total lipid (%) content of *Tetraselmis striata* in axenic conditions (T) with different treatments of marine broth (MB) and *Pelagibaca bermudensis* exudates (t) in O3 medium. The values are presented in mean ± SD (*n* = 3). Error bars are showing SD. Post hoc analysis is shown in the Additional file 2: Table S1 (G, H) for comparing the means according to their significant difference (LSD)

The total lipid contents of T-PB under limited sulfur, metal, and nitrate conditions were 7.65 ± 0.08, 15.34 ± 0.62, and 32.47 ± 0.24% with biomass productivities of 18.93 ± 1.15, 10.97 ± 0.77, and 16.48 ± 1.18 mg L^−1^ day^−1^, respectively, while the total lipid contents under T conditions with limited sulfate, metal, and nitrate were 7.34 ± 0.48, 20.17 ± 0.82, and 31.47 ± 0.2% with biomass productivities of 10.60 ± 1.15, 7.45 ± 1.88, and 2.08 ± 0.72, mg L^−1^ day^−1^, respectively (Fig. 4). The sulfate limitation reduced the lipid content, but it maximized the biomass productivity most among the nutrient limitation conditions. Sulfur limitation in *Tetraselmis* has been reported to cause higher accumulation of starch than lipid [47]. The higher biomass productivity under co-cultivated conditions compared to axenic conditions indicated that the *P. bermudensis* involved in multi-functional co-operation to the *T. striata*. Furthermore, of the nutrient limitation conditions, nitrate limitation enhanced the lipid content, while the sulfur and trace metal limitations reduced the lipid content. Moreover, only nitrate limitation induced lipid accumulation, which showed a 1.46–1.55-fold higher lipid content than that under the nutrient replete condition (O3-control) and, therefore, was selected for the two-stage cultivation in the current study (Fig. 4).

However, all nutrient limited conditions restricted the growth of *T. striata* in the presence and absence of *P. bermudensis*. *T. striata* cells exhibited a higher survival rate in the presence of *P. bermudensis* than under T conditions (Additional file 1: Figure S5). The trace metal might have been regenerated by the re-mineralization process of the decaying cells or the metalloproteins/metallopeptides, or both [48]. The hypothesis proposed in an independent study on DMSP degradation by the *Roseobacter* clade bacteria using HHQ [44] could be one of the strategies to address the partial requirement of sulfur for *T. striata*. Therefore, the microalgal cell abundance and biomass productivity were higher under limited sulfur levels than under axenic *T. striata* cultivation with limited sulfur levels (Fig. 4 and Additional file 1: Figure S5).

### Effect of exo-metabolites and mixotrophy

**Fig. 5:**
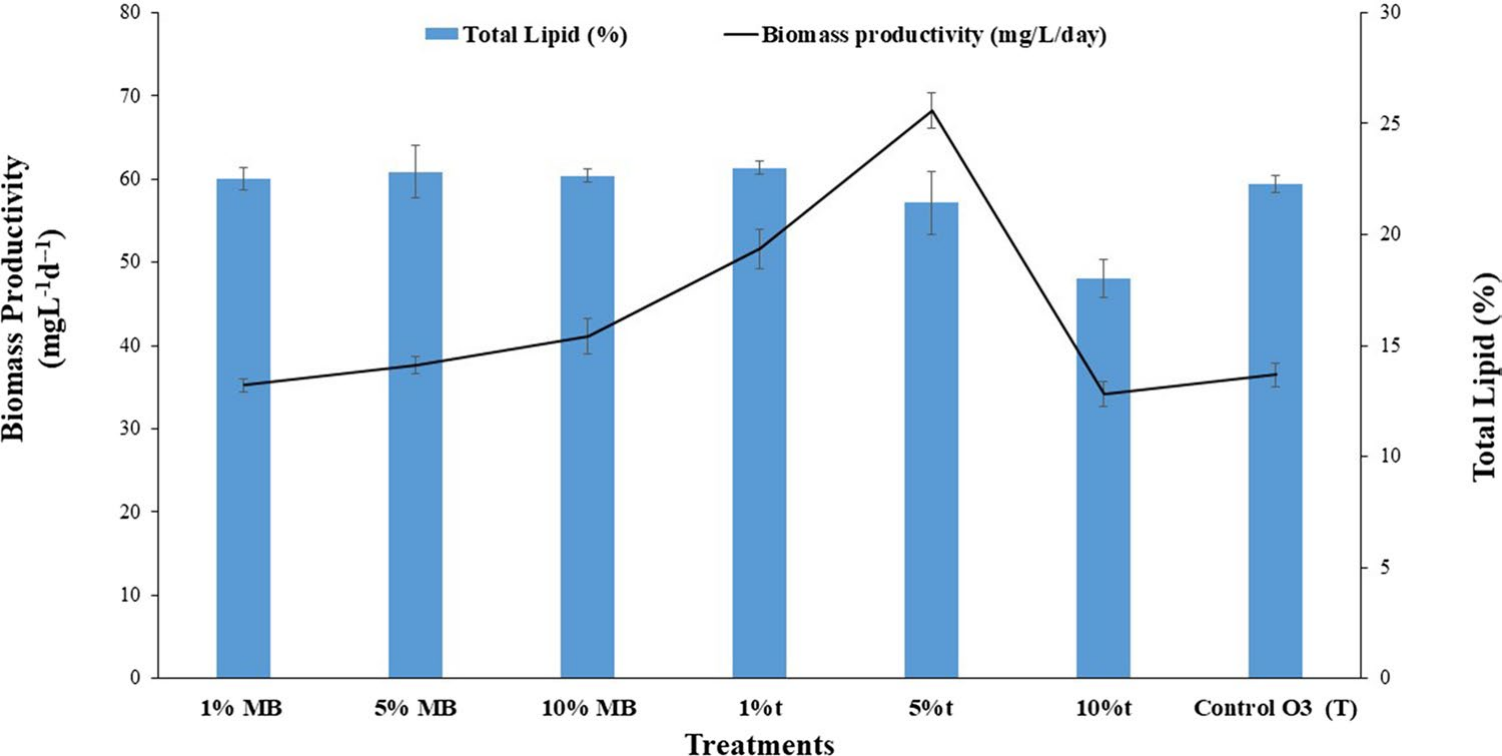
Biomass productivity, lipid productivity, and total lipid content (%) of *Tetraselmis striata* in axenic (T) and co-culture (T-PB) conditions under nutrient limitation (sulfate, nitrate, and trace metal), nutrient replete, and two-stage cultivation mode (salinity increase, nitrate limitation, and decreased pH). The values are presented in mean ± SD (*n* = 3). Error bars are showing SD

**Fig. 6:**
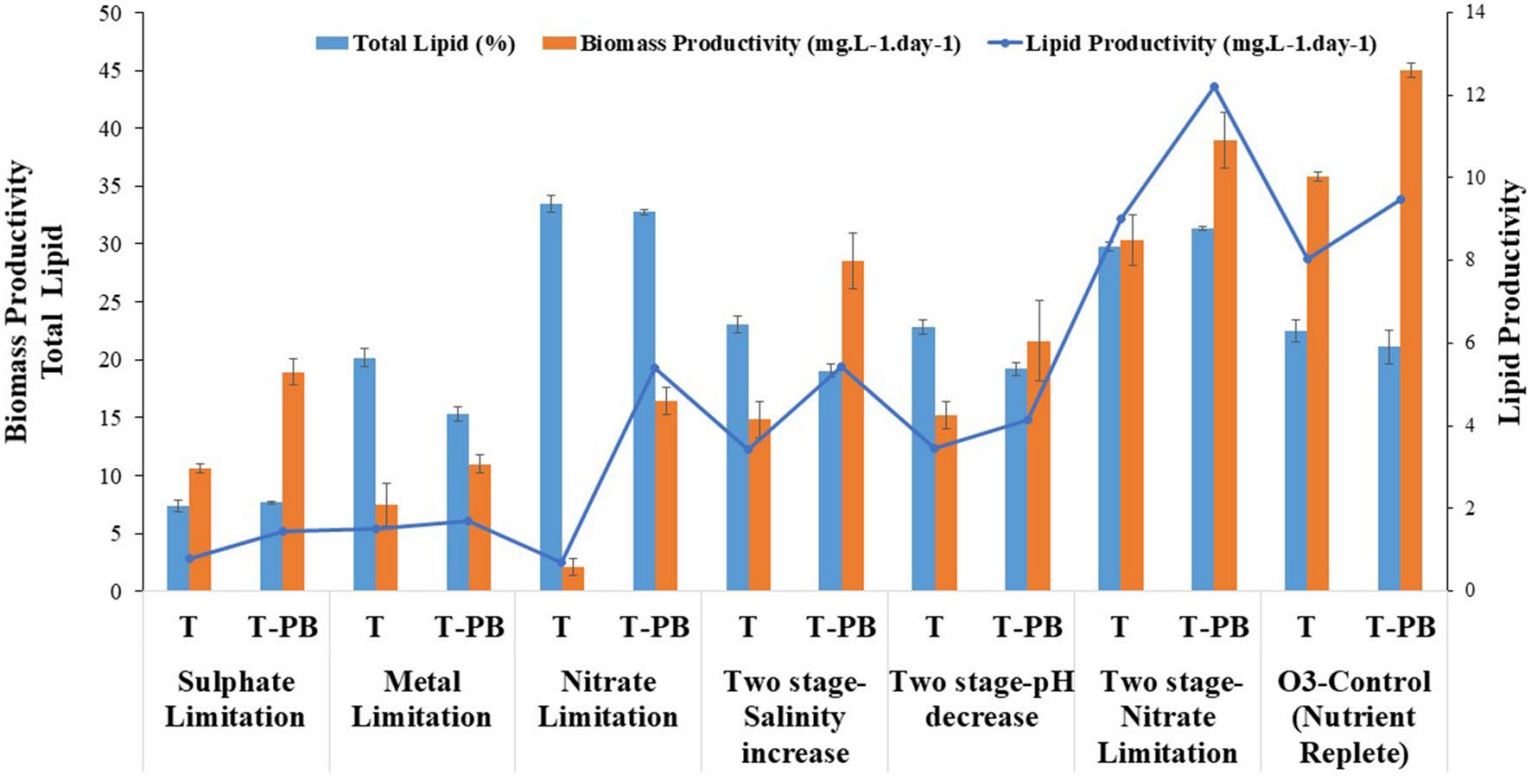
Cell abundance of *T. striata* in varying *P. bermudensis* exudates (t) and marine broth exudates (MB) in O3 media. The values are presented in mean ± SD (*n* = 3). Error bars are showing SD

The crude exudates containing metabolites released by *P. bermudensis* were shown to elevate the biomass productivity by up to 41.63 and 91.73% at 1 and 5% of exudates, respectively (Fig. 5). Furthermore, 1 and 5% exo-metabolite treatment increased the biomass of the *T. striata* successively, while the 10% treatment did not enhance the growth as reflected in the reduced cell abundance in the stationary phase compared to the MB-treated cultures (Fig. 6). This indicated that the *T. striata* could not cope with the higher metabolite concentration of *P. bermudensis*. The results indicate that the physical presence of *P. bermudensis* was not essential for the growth promoting effect, and the metabolites released by *P. bermudensis* were closely involved in promoting the microalgal growth. The microalgal cells did not grow in 100% exudate of *P. bermudensis* and MB (t), and the 100% MB-containing medium (without exudates of *P. bermudensis*) showed that *T. striata* did not grow mixotrophically or heterotrophically under these conditions.

The HHQ (10 µM) and PQS (1000 µM) individually exhibited algicidal effect on the axenic *T. striata* (Additional file 1: Figure S6). These precursors are beneficial in formation of biofilm of *P. bermudensi*s on the cell wall of *T. striata* for mutualistic growth but increased concentration control the growth of *T. striata*. In addition, the exudates released by *P. bermudensis* containing the cocktail of growth promoting metabolites as well as algicidal compounds, so the net effect of the metabolites was growth promoting (Fig. 5).

However, the culture, which was exposed to only MB in the O3 medium (without the exudates of *P. bermudensis*), also exhibited enhanced growth under successive concentration (5 and 10% MB in O3) due to the mixotrophic capability of the *T. striata*; however, the rate was less than that of the exudate-treated cultures (t). MB also contains organic substrates and nutrients. A higher organic substrate concentration (100% MB and 100% t) elevates the intracellular CO_2_ level and, hence, causes toxicity to microalgae [9]. Furthermore, 5% was the optimum condition with a 68.28 ± 2.12 mg L^−1^ day^−1^ biomass productivity and 21.43 ± 1.41% lipid content. Therefore, this condition is lucrative for the scaleup in wastewater/saline effluent as well as in seawater (Fig. 5). Mixotrophic cultivation is an impressive strategy for wastewater remediation where high organic load containing various organic substrates with inorganic nutrients would be available to synergize the maximum biomass production [49–51]. Furthermore, biomass productivity would be even higher under sunlight conditions, since the exudates enhanced mixotrophic growth employed in the current experiment had only 45 µM m^−2^ s^−1^. Furthermore, to scale up the cultivation of *T. striata,* extensive investigation on mechanisms of growth stimulating and growth inhibiting allelopathic compounds is essential, at lab, pilot, and commercial production scales [52].

### Amelioration of biomass productivity and lipid content using two-stage cultivation

The two-stage cultivation (15 day, first stage; 3 day, second stage) technique was performed in an attempt to ameliorate the lipid content. The biochemical composition and precursors were modulated by the abrupt changes in the medium and physical environment, which decreased the microalgal cell abundance. The *salinity increase* in the two-stage cultivation mode exhibited a biomass productivity of 14.81 ± 1.52 and 28.52 ± 2.39 mg L^−1^ day^−1^ with 23.04 ± 0.71 and 19.04 ± 0.58% lipid content under T and T-PB conditions, respectively. Furthermore, the *pH decrease* (low pH, 6.0) in the stage II resulted in a biomass productivity of 15.23 ± 1.16 and 21.62 ± 3.44 mg L^−1^ day^−1^ with a lipid content of 22.78 ± 0.39 and 19.20 ± 0.17%, respectively (Fig. 4). The nitrate limitation in the two-stage cultivation resulted in a biomass productivity of 30.28 ± 2.18 and 38.95 ± 2.45 mg L^−1^ day^−1^ with an increased lipid content of 29.75 ± 0.97 and 31.37 ± 1.46% under T and T-PB conditions, respectively (Fig. 4). Salinity and pH in the two-stage mode did not result in higher lipid content with a more impressive biomass productivity than the control (nutrient replete condition).

The strategic stress induced for the 3 days could not be optimum, but the results of this study support the usefulness of the two-stage cultivation model for *T. striata* under both T and T-PB conditions using nitrate limitation. The optimum stress induced by the elevated salinity/temperature/nutrient limitation mediated biochemical macromolecular synthesis, which would modulate metabolism to induce the levels of carbohydrate or lipid accumulation in *Tetraselmis*; however, the response varies within genera/species [10, 18, 19, 47]. Optimum stress conditions in two-stage cultivation for the amelioration of lipid productivity have been previously demonstrated in *Tetraselmis*, *Scenedesmus*, *Acutodesmus*, *Nannochloropsis*, *Chlorella*, *Synechococcus,* and *Monoraphidium* and *Chlamydomonas* [9, 19, 53–57] where the type, time, and intensity of the stress matter. Two-stage cultivation could enhance potential of overall biofuel production process by increasing the desirable biochemical feedstock. Furthermore, it creates an opportunity to use the enormous potential of varied effluents strategically, while providing nutrients and a suitable physical environment for attaining the maximum lipid productivity [50, 51, 57]. The growth promotion effect shown in some of the optimized conditions for higher biomass productivity and lipid productivity are in agreement with the previous studies performed in other co-cultivation strategies. However, the increment folds in lipid yields compared to the axenic condition were higher in the immobilized conditions in *Chlorella* spp. (Table 2) [58–66].

**Table 2:** Comparison of co-cultivation of microalgae with bacteria to their axenic/control conditions for the improvement in biomass and growth

Co-cultivation	Fold increase in biomass productivity, growth rate, biomass, or cell size	Media	References
*Chlamydomonas reinhardtii*, *Chlorella vulgaris,* and *Scenedesmus* with growth promoting bacteria	Increment of specific growth rate of 1.13-fold in *C. reinhardtii*, 1.08-fold in *C. vulgaris* and 1.10-fold in *Scenedesmus* sp.	BG11 and TAP medium at 30 °C for 12 days with a light intensity of 50 μE m^−2^ s^−1^	[58]
*Chlorella vulgaris* with *Rhizobium* and *Flavobacterium*	1.92–2.55-fold increment in cell mass. 1.25-fold increment in lipid content	BG-11 (constant stirring at 100 rpm, 25 °C, light intensity of 100 μmol m^−2^ s^−1^)	[59]
*Chlorella vulgaris* with beneficial bacteria *Duganella* sp. JPPB B33 (GU368376) and *Pseudomonas putida* strain MM1 (AY623928) with some other uncultured bacteria	Both increment and decrement of biomass productivity due to the influence of uncultured bacteria	Wastewater medium with varying bacterial treatments	[60]
*Ankistrodesmus* and *Rhizobium*	1.29-fold increment in biomass concentration in bubble photobioreactor. 1.19-fold increment in airlift photobioreactor (dry weight)	AEX medium; in air bubble photobioreactor	[61]
*Botryococcus braunii* and *Candidatus Phycosocius bacilliformis*	1.8-fold in the biomass and 1.5-fold improvement in total hydrocarbon yield	Vitamin containing modified media of TSP	[62]
*Microbacterium* sp. and *Chlorella vulgaris*	1.66-fold increment in biomass (cell dry weight)	Modified MBG medium	[63]
*Acidovorax facilis* and *Scenedesmus obliquus*	1.24-fold in biomass productivity and 1.29-fold in lipid productivity	BG 11 medium	[64]
*Chlorella* and *Azospirillum brasilense*	Increased the cell size up to 1.62-fold in *Chlorella vulgaris*, and total lipid yield increased up to 1.5–3.8 times in varying *Chlorella* spp.	Sterile mineral medium (C30) and co-cultivated in the alginate beads	[65]
*Chlorella* and *Azospirillum brasilense*	2.1-fold in fresh weight and 24-fold increase in the dry weight of alginate beads containing the per gram of microbial biomass	Residual water medium (synthetic wastewater medium) and co-cultivated in alginate beads	[66]
*Stappia* sp., *Pelagibaca bermudensis* and *Tetraselmis striata*	1.5–2-fold improvement in biomass productivity	O3 Media with higher *T. striata* concentration as inoculation	[12]
*P. bermudensis* and *T. striata*	1.2–3-fold improvement in biomass productivity in varying stressors; 1.5-fold improvement in lipid productivity	O3 media with varying physicochemical conditions	This study

The results obtained in the current study, indicating that the microalgal biofuel co-cultivation model would be applicable for the range of environmental variables. However, the open pond cultivation would have several climatic factors as well as biotic factors. The dominant biotic stressor and interaction of *P. bermudensis* with the native seawater bacterial communities needed to be investigated in more details. Optimization of the inoculum density of *P. bermudensis* as well as the search for the other co-operating bacteria would be essential to overcome with the competition of seawater native bacterial communities.

The previous studies done by our group [12] to evaluate the feasibility of this model with native seawater bacteria indicated that the cell abundance of *P. bermudensis* (10^5^) was incapable to outcompete the seawater bacterial communities, but growth promotion was also seen under the adverse conditions. This was probably due to metabolic products released by the growth promoting probiotic bacteria. The effect shown by the cell culture filtrate treatment of *P. bermudensis* (t) on *T. striata* confirmed this hypothesis that metabolic exudates of the *P. bermudensis* could show growth promoting effect. The large-scale open pond cultivation should be investigated to unravel the exo-metabolomic dynamics (growth hormones as well as algicidal compounds released by the native seawater or saline water communities) to exactly pinpoint the mechanisms and realize the application.

It would be an interesting methodological question that which growth phase of *T. striata* and *P. bermudensis* should be employed to see the mutualistic interaction in addition to varying stressors (as variables) in different culture mode. Furthermore, the interactions studies in varying co-cultivation in light of *multi* omics approaches will certainly unfold the vivid transcriptomic and metabolic changes, real-time growth promoting effects, and metabolic nutrients trade-off between the organisms [67, 68].

## Conclusion

Co-cultivation of *T. striata* with *P. bermudensis* could be a potentially robust and sustainable technique for biomass generation using a wide spectrum of abiotic parameters with ameliorated stress tolerance. *P. bermudensis* also demonstrated a growth promoting effect on *T. striata*. Two-stage cultivation using nitrate limitation and continuous high-light exposure with optimum salinity (20 g L^−1^) at pH 8.0, as well as metabolic feed of *P. bermudensis* is suggested as a useful strategy for attaining higher lipid productivity of *T. striata*. Exo-metabolomics would certainly unfold the vivid interactions between microalgae and bacteria, and would elucidate the specific role of growth promoting metabolites and their implications for ensuring lipid productivity.

## Additional files

**Additional file 1: Figure S1.** Cell abundance of *Pelagibaca bermudensis* in co-cultivation (*Tetraselmis striata*–*P. bermudensis*; T-PB) culture during the different interval of the growth (at different pH). **Figure S2.** Cell abundance of *Pelagibaca bermudensis* in co-cultivation (*Tetraselmis striata*–*P. bermudensis*; T-PB) culture during the different interval of the growth (at different salinity). **Figure S3.** Cell abundance of *Tetraselmis striata* in axenic (T) and co-cultivated (T-PB) growth mode at different photoperiods (12:12 and 24:00 hrs with 147 µM m^−2^ s^−1^). **Figure S4.** Cell abundance of *Pelagibaca bermudensis* in co-cultivation (*Tetraselmis striata*–*P. bermudensis;* T-PB) culture during the different interval of the growth (at different temperature and light conditions). **Figure S5.** Cell abundance of *Tetraselmis striata* (T) exposed to varied HHQ and PQS concentration and controls (without HHQ and PQS; with equal nontoxic acetonitrile). **Figure S6.** Cell abundance of *Tetraselmis striata* in axenic (T) and co-cultivated (T-PB) growth mode at different nutrient limited and replete conditions in O3 media.

**Additional file 2: Table S1.** Post hoc *t* analysis for comparison of means (LSD) based on one-way ANOVA results of the data shown in Figs. 2 and 4 (Biomass productivity and total lipid content under different environmental variables and two-stage cultivation); (Table A–H).

## Abbreviations

HHQ: 4-hydroxy-2-alkylquinolines
PQS (Pseudomonas quorum signal): 2-heptyl-3-hydroxy-4(1H)-quinolone
T: axenic condition
T-PB: co-cultivation condition
