# Correction to: *Pelagibaca bermudensis* promotes biofuel competence of *Tetraselmis striata* in a broad range of abiotic stressors: dynamics of quorum-sensing precursors and strategic improvement in lipid productivity

**DOI:** 10.1186/s13068-018-1185-x

**Published:** 2018-07-03

**Authors:** Shailesh Kumar Patidar, Sae-Hee Kim, Jin Ho Kim, Jungsoo Park, Bum Soo Park, Myung‑Soo Han

**Affiliations:** 1 Department of Life Science, College of Natural Sciences, Hanyang University, Seoul, South Korea; 2 Research Institute of Natural Sciences, Hanyang University, Seoul, South Korea; 3 Present Address: Marine Science Institute, University of Texas at Austin, Port Aransas, TX USA

## Correction to: Biotechnol Biofuels (2018) 11:102 10.1186/s13068-018-1097-9

After publication of the original article [1], it was brought to our attention that Figs. 4, 5, and 6 and their captions were incorrect. The correct figures and captions are presented below:

**Fig. 4:**
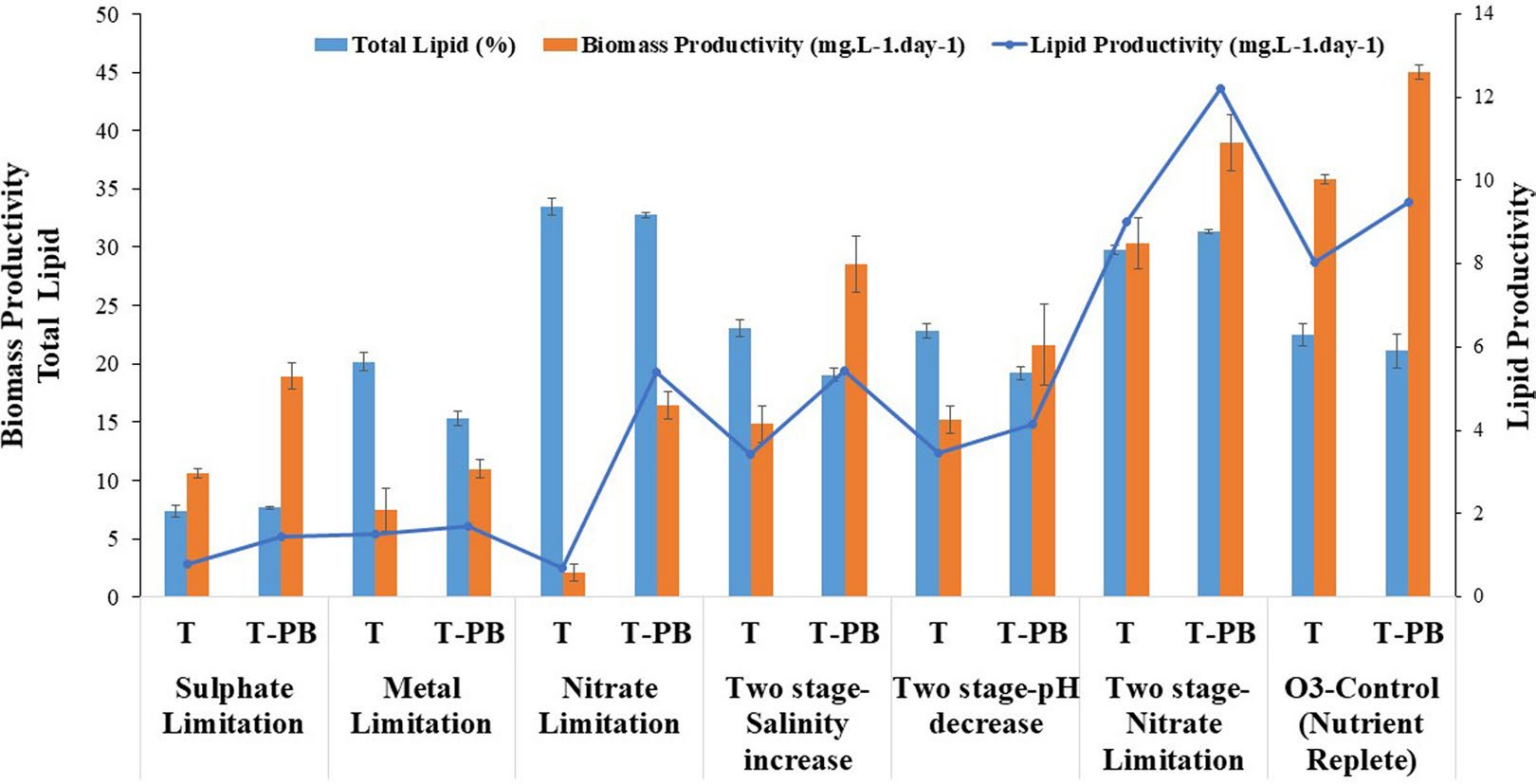
Biomass productivity and total lipid (%) content of *Tetraselmis striata* in axenic conditions (T) with different treatments of marine broth (MB) and *Pelagibaca bermudensis* exudates (t) in O3 medium. The values are presented in mean ± SD (n = 3). Error bars are showing SD. Post hoc analysis is shown in the Additional file 2: Table S1 (G, H) for comparing the means according to their significant difference (LSD)

**Fig. 5:**
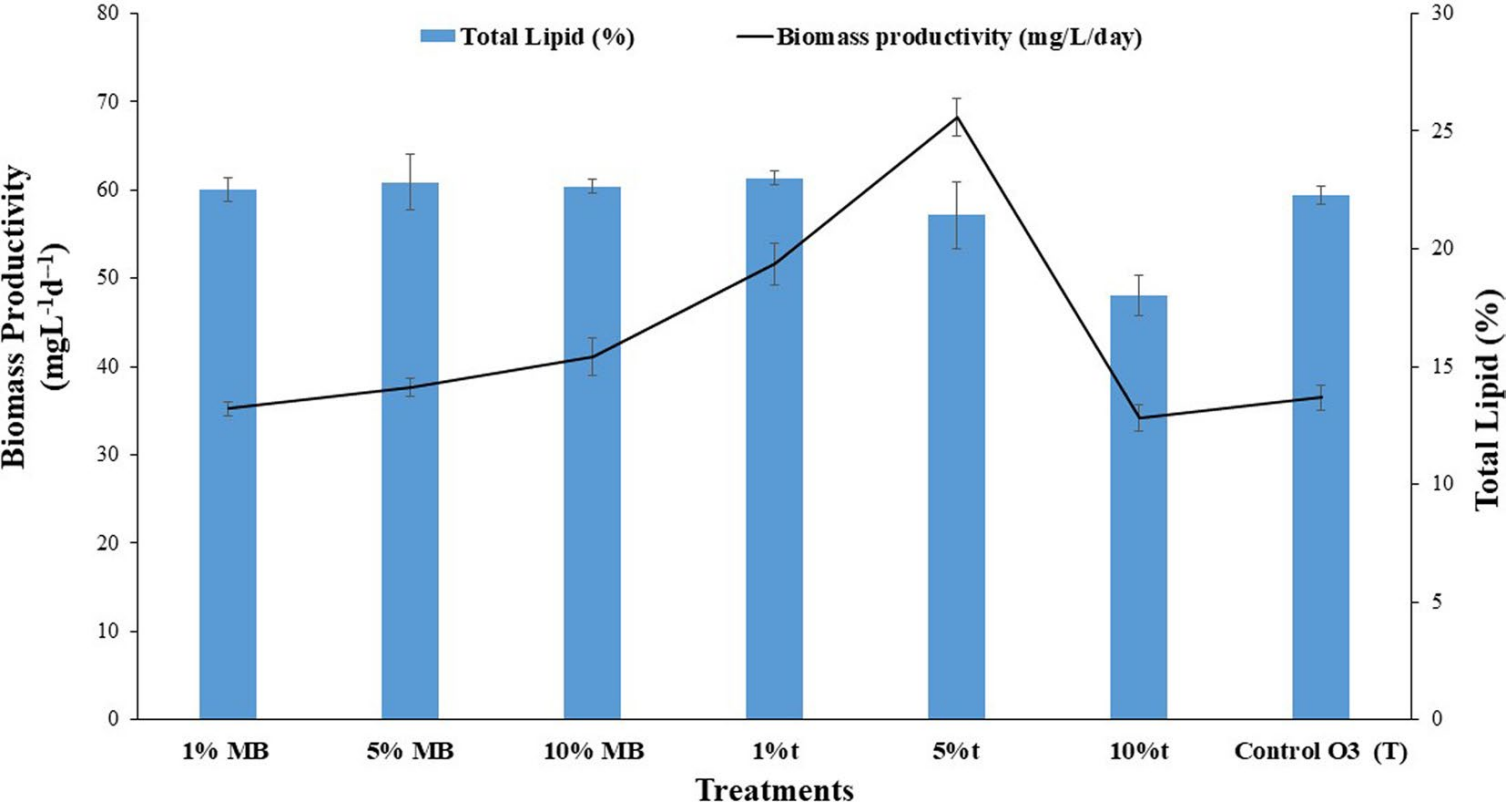
Biomass productivity and total lipid content of *Tetraselmis striata* under different exudates treatments. *MB *=* Marine broth exudates, t *=* Exo*-*metabolites of P. bermudensis *+* Marine broth*

**Fig. 6:**
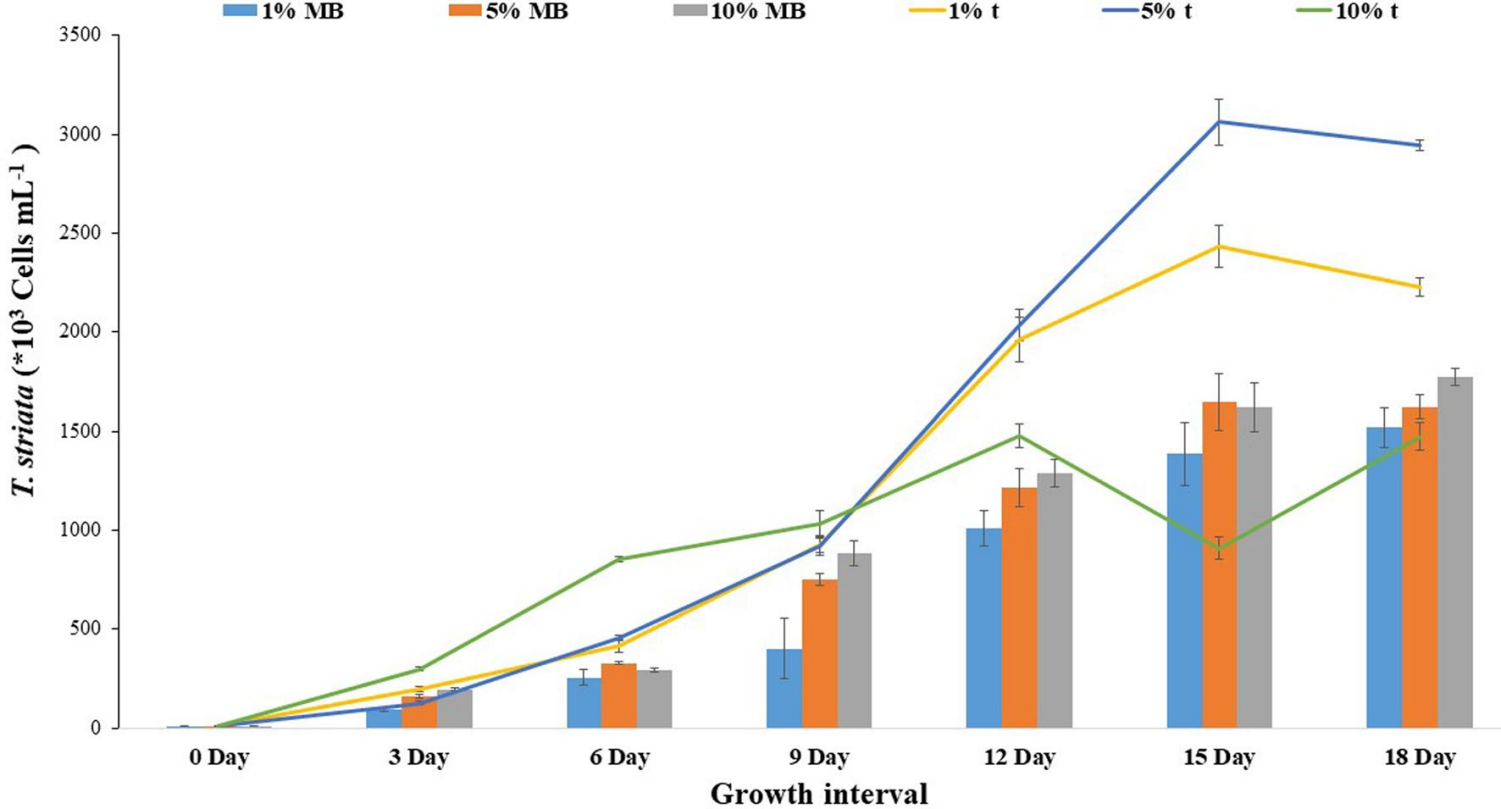
Cell abundance of *T. striata* in varying *P. bermudensis* exudates (t) and marine broth exudates (MB) in O3 media. The values are presented in mean ± SD (n = 3). Error bars are showing SD

Table 1 also contained an error. The column title ‘Light (µM cm ^− 2^ S ^− 1^)’ should instead be ‘Light (µM m^− 2^ S ^− 1^)’.

In the ‘Results and discussion’ session the following sentence is incorrect:

‘In fact, the dead cells could be degraded by the HHQ and the degradation complex molecules were probably simpler growth metabolites that could be used by the active cells of *T. striata* and *P. bermudensis*. HHQ is also known to degrade dimethylsulfoniopropionate (DMSP) [44].’

The correct sentence is as follows:

‘In fact, the dead cells could be degraded by the *P. bermudensis* and the degradation complex molecules were probably simpler growth metabolites that could be used by the active cells of *T. striata* and *P. bermudensis*. *P. bermudensis* is also known to degrade dimethylsulfoniopropionate (DMSP) [44].’
