# Supplementary material for: Pelagibaca bermudensis promotes biofuel competence of Tetraselmis striata in a broad range of abiotic stressors: dynamics of quorum-sensing precursors and strategic improvement in lipid productivity
Source: Biotechnol Biofuels. 2018 Apr 7;11:102. doi: 10.1186/s13068-018-1097-9 (PMC5889607; doi:10.1186/s13068-018-1097-9)
Supplement: Supplementary file 1 — Additional file 1: Figure S1. Cell abundance of Pelagibaca bermudensis in co-cultivation (Tetraselmis striata–P. bermudensis; T-PB) culture during the different interval of the growth (at different pH). Figure S2. Cell abundance of Pelagibaca bermudensis in co-cultivation (Tetraselmis striata–P. bermudensis; T-PB) culture during the different interval of the growth (at different salinity). Figure S3. Cell abundance of Tetraselmis striata in axenic (T) and co-cultivated (T-PB) growth mode at different photoperiods (12:12 and 24:00 hrs with 147 µM m−2 s−1). Figure S4. Cell abundance of Pelagibaca bermudensis in co-cultivation (Tetraselmis striata–P. bermudensis; T-PB) culture during the different interval of the growth (at different temperature and light conditions). Figure S5. Cell abundance of Tetraselmis striata (T) exposed to varied HHQ and PQS concentration and controls (without HHQ and PQS; with equal nontoxic acetonitrile). Figure S6. Cell abundance of Tetraselmis striata in axenic (T) and co-cultivated (T-PB) growth mode at different nutrient limited and replete conditions in O3 media. [file 13068_2018_1097_MOESM1_ESM.pptx]

## Slide 1
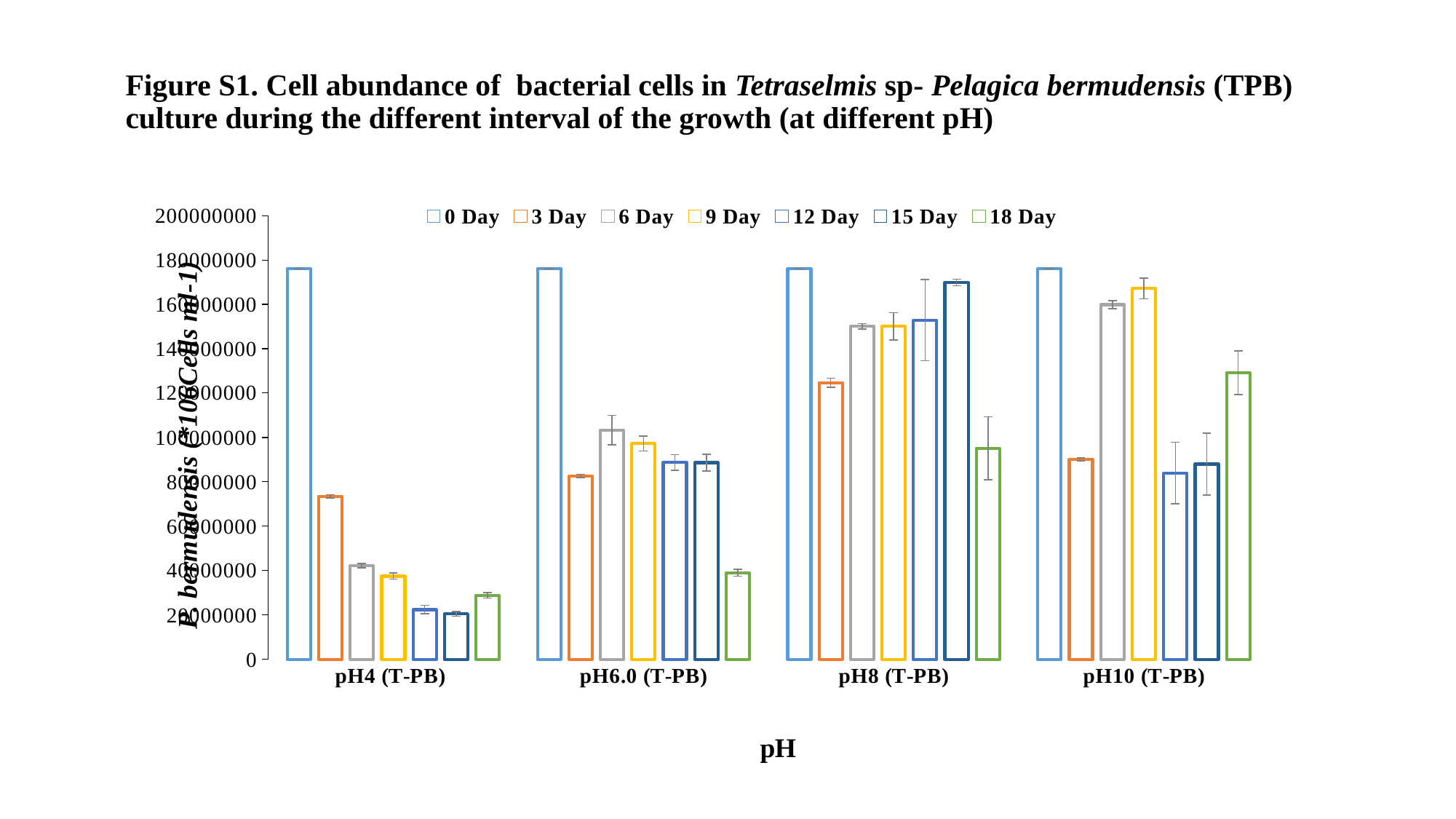

# Figure S1. Cell abundance of bacterial cells in Tetraselmis sp- Pelagica bermudensis (TPB) culture during the different interval of the growth (at different pH)
### Chart
| Category | 0 Day | 3 Day | 6 Day | 9 Day | 12 Day | 15 Day | 18 Day |
|---|---|---|---|---|---|---|---|
| pH4 (T-PB) | 176015800.0 | 73513268.09400116 | 42234786.23669781 | 37522397.256313086 | 22442752.519082084 | 20557796.9269282 | 28922287.36711102 |
| pH6.0 (T-PB) | 176015800.0 | 82702426.60575122 | 103378033.25718915 | 97251927.58268903 | 88769627.41799663 | 88651817.69348708 | 39053923.67493811 |
| pH8 (T-PB) | 176015800.0 | 124662323.48525997 | 150089589.02525237 | 150089589.02525237 | 152917022.41348308 | 169881622.74286798 | 95131352.5415159 |
| pH10 (T-PB) | 176015800.0 | 90124439.24985719 | 159867796.1595506 | 167113094.21689203 | 84003864.2086841 | 88003864.20868407 | 129207815.35592262 |

## Slide 2
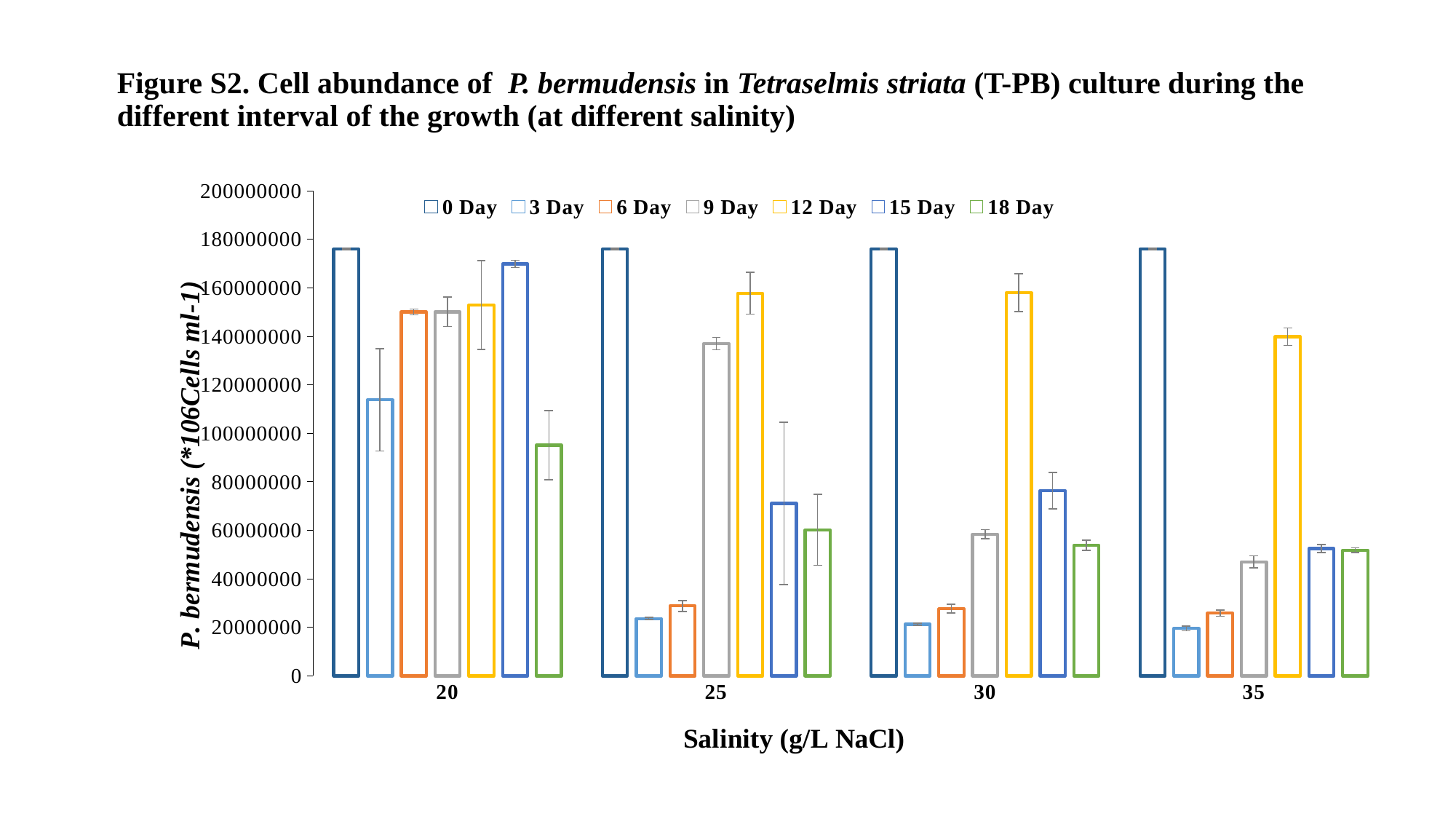

# Figure S2. Cell abundance of P. bermudensis in Tetraselmis striata (T-PB) culture during the different interval of the growth (at different salinity)
### Chart
| Category | 0 Day | 3 Day | 6 Day | 9 Day | 12 Day | 15 Day | 18 Day |
|---|---|---|---|---|---|---|---|
| | 176015800.0 | 113804193.87629025 | 150089589.02525237 | 150089589.02525237 | 152917022.41348314 | 169881622.74286804 | 95131352.5415159 |
| | 176015800.0 | 23667973.653982107 | 28863382.504856225 | 137042162.0358123 | 157816354.034965 | 71157073.60380882 | 60259674.08666923 |
| | 176015800.0 | 21323560.13624072 | 27714737.69088745 | 58433623.35677014 | 157981041.302904 | 76399606.34448679 | 53839044.10089508 |
| | 176015800.0 | 19568195.241047416 | 25888686.96098838 | 47064984.9415921 | 139869595.4240431 | 52543137.1312893 | 51836278.7842316 |

## Slide 3
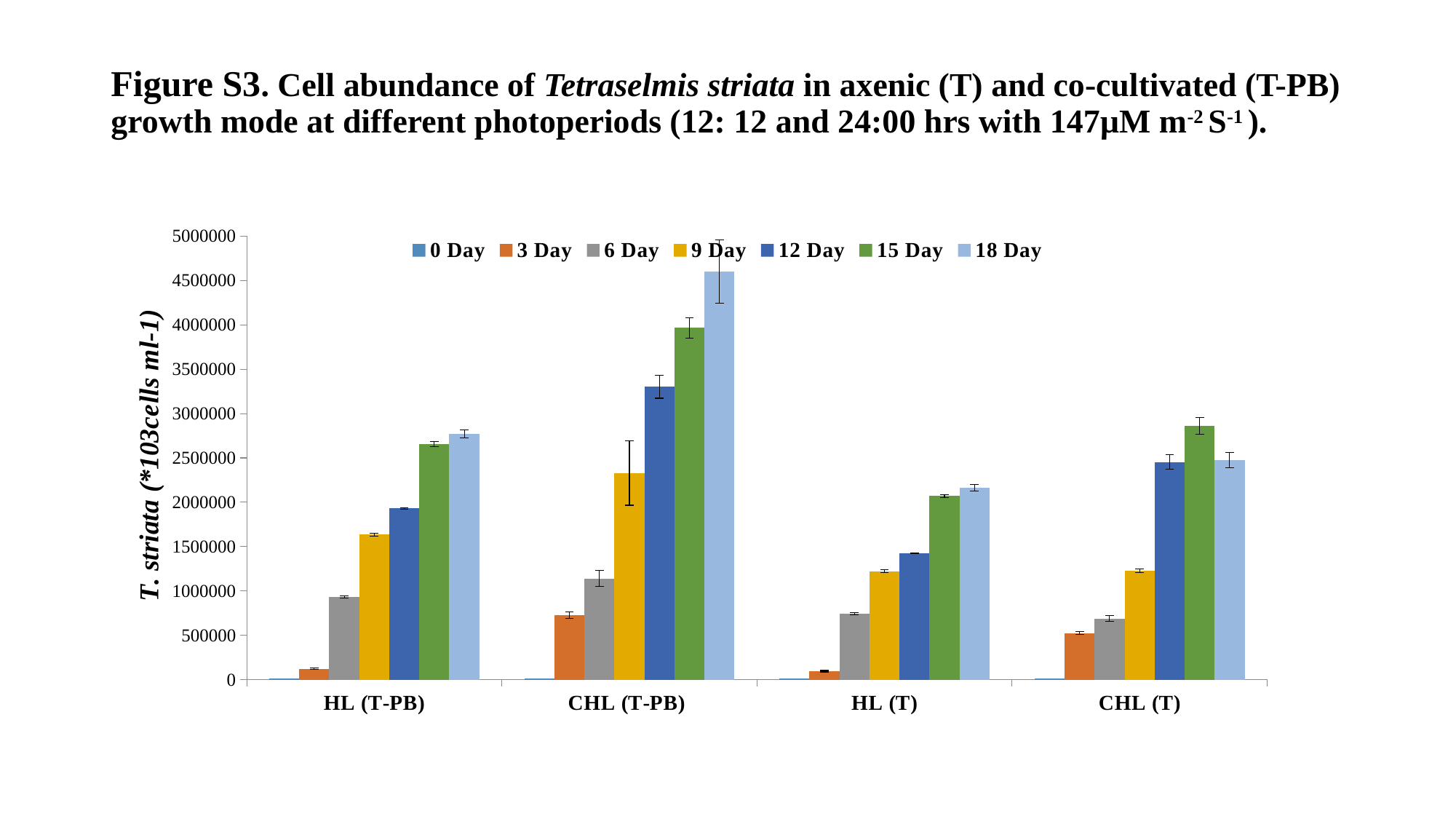

# Figure S3. Cell abundance of Tetraselmis striata in axenic (T) and co-cultivated (T-PB) growth mode at different photoperiods (12: 12 and 24:00 hrs with 147µM m-2 S-1 ).
### Chart
| Category | 0 Day | 3 Day | 6 Day | 9 Day | 12 Day | 15 Day | 18 Day |
|---|---|---|---|---|---|---|---|
| HL (T-PB) | 10000.0 | 122500.0 | 932500.0 | 1634166.666666667 | 1932500.0 | 2655833.3333333326 | 2770833.3333333326 |
| CHL (T-PB) | 10000.0 | 730000.0 | 1140000.0 | 2329166.6666666665 | 3302500.0 | 3965000.0 | 4600000.0 |
| HL (T) | 10000.0 | 95833.33333333333 | 744166.6666666667 | 1221666.666666667 | 1428333.3333333333 | 2070000.0 | 2161666.6666666665 |
| CHL (T) | 10000.0 | 525000.0 | 687500.0 | 1226666.666666667 | 2453333.3333333326 | 2859166.6666666665 | 2475000.0 |

## Slide 4
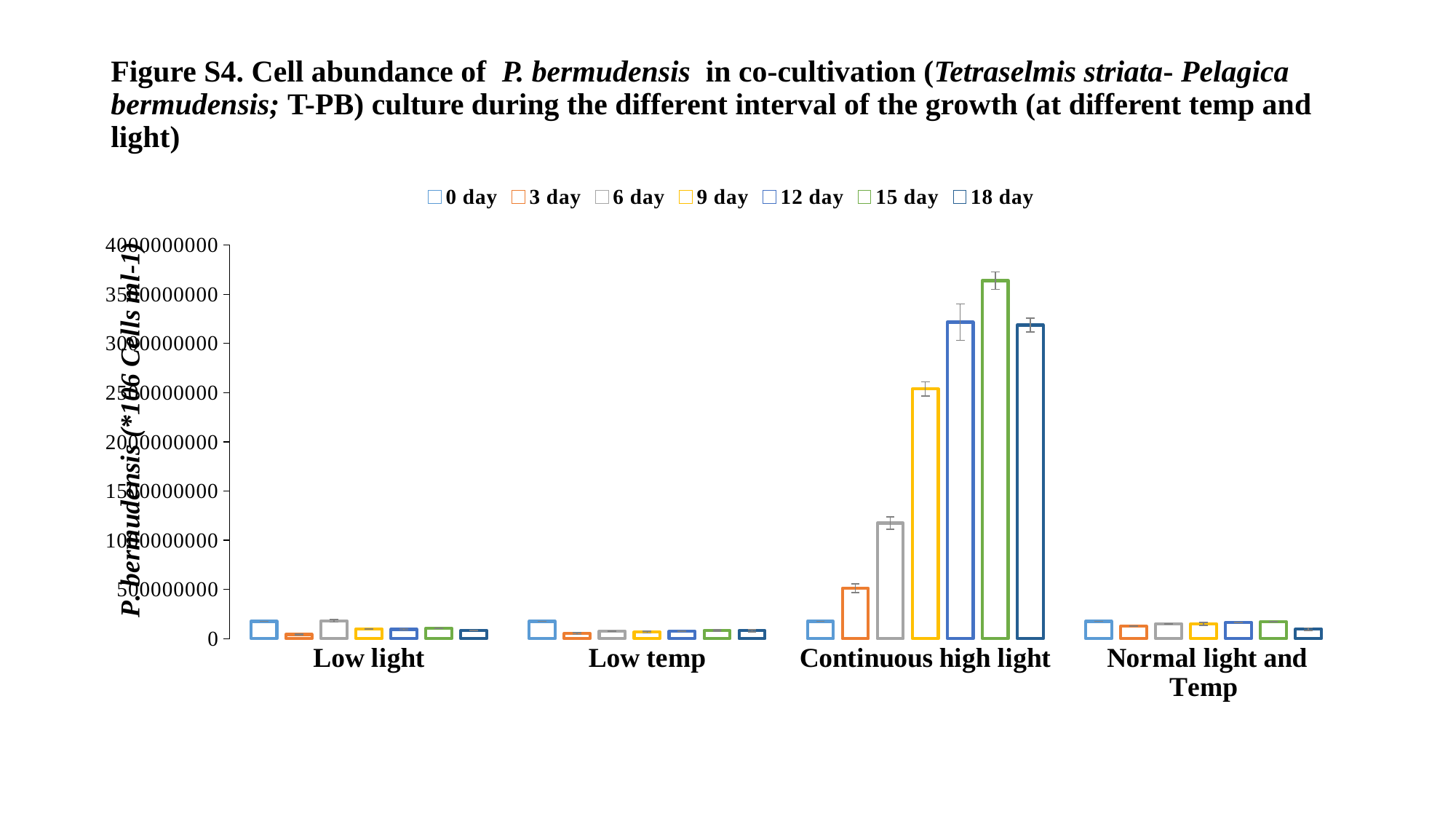

# Figure S4. Cell abundance of P. bermudensis in co-cultivation (Tetraselmis striata- Pelagica bermudensis; T-PB) culture during the different interval of the growth (at different temp and light)
### Chart
| Category | 0 day | 3 day | 6 day | 9 day | 12 day | 15 day | 18 day |
|---|---|---|---|---|---|---|---|
| Low light | 176015800.0 | 42588215.410226636 | 180837927.12226248 | 99549217.2106266 | 93894350.43416494 | 104791749.95130455 | 82290092.56996766 |
| Low temp | 176015800.0 | 51836278.78423159 | 75398223.68615502 | 65973445.725385666 | 74161221.57880405 | 82231187.70771283 | 79639373.76850124 |
| Continuous high light | 176015800.0 | 511294204.37173873 | 1173973904.7383356 | 2538210514.5597034 | 3216205479.112551 | 3638553341.479529 | 3186753047.9851475 |
| Normal light and Temp | 176015800.0 | 124760498.25568464 | 150089589.025252 | 150089589.02525237 | 164580185.1399353 | 169881622.74286804 | 95131352.5415159 |

## Slide 5
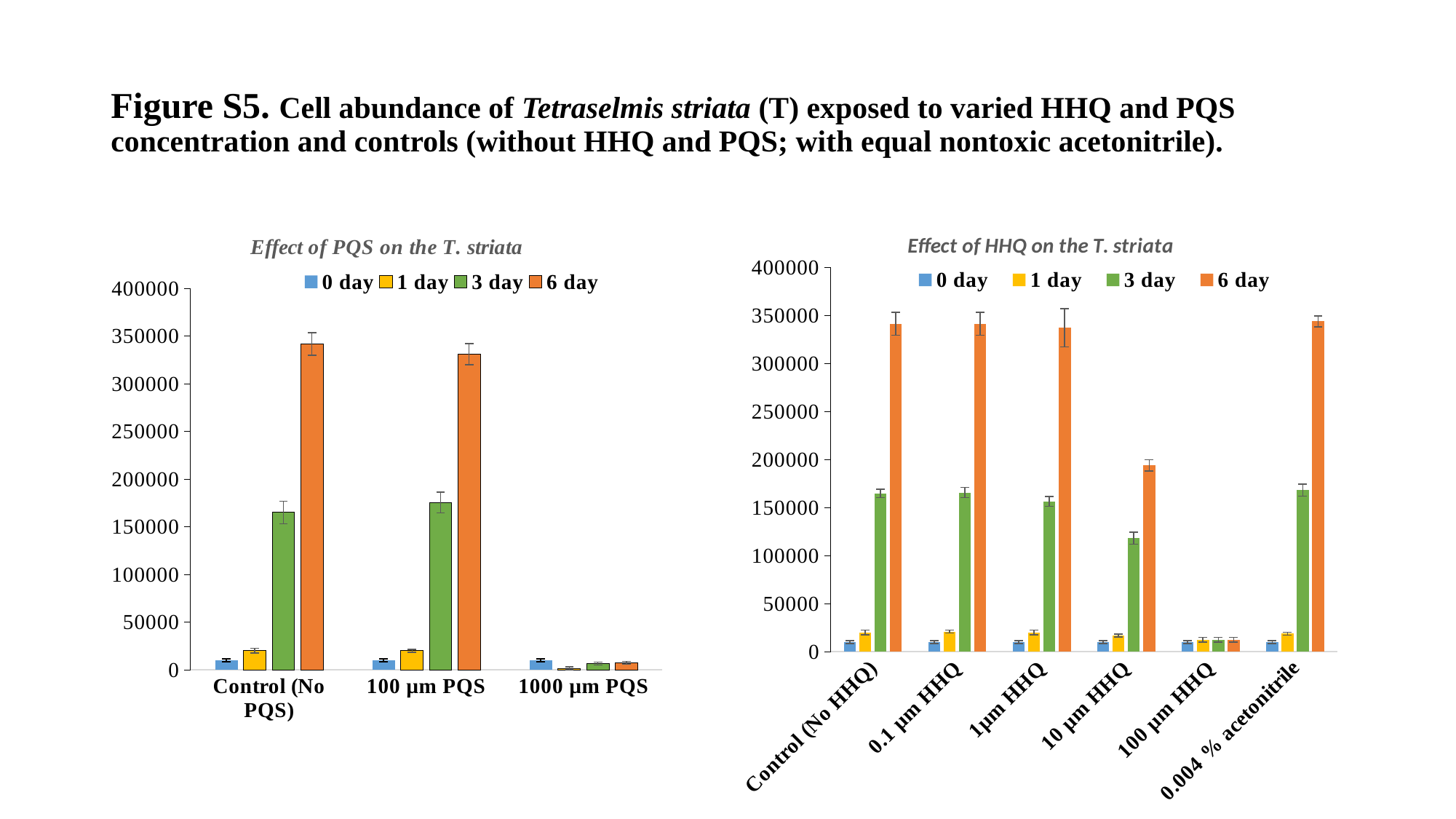

# Figure S5. Cell abundance of Tetraselmis striata (T) exposed to varied HHQ and PQS concentration and controls (without HHQ and PQS; with equal nontoxic acetonitrile).
### Chart: Effect of PQS on the T. striata
| Category | 0 day | 1 day | 3 day | 6 day |
|---|---|---|---|---|
| Control (No PQS) | 10000.0 | 20000.0 | 165000.0 | 341666.6666666667 |
| 100 µm PQS | 10000.0 | 19890.0 | 175500.0 | 331000.0 |
| 1000 µm PQS | 10000.0 | 1443.3756729740646 | 6666.666666666702 | 7500.0 |
### Chart: Effect of HHQ on the T. striata
| Category | 0 day | 1 day | 3 day | 6 day |
|---|---|---|---|---|
| Control (No HHQ) | 10000.0 | 20000.0 | 165000.0 | 341666.6666666667 |
| 0.1 µm HHQ | 10000.0 | 20833.33333333333 | 165833.3333333333 | 341666.6666666667 |
| 1µm HHQ | 10000.0 | 20000.0 | 156666.66666666666 | 337500.0 |
| 10 µm HHQ | 10000.0 | 16666.666666666664 | 118333.33333333333 | 194166.66666666666 |
| 100 µm HHQ | 10000.0 | 12500.0 | 12500.0 | 12500.0 |
| 0.004 % acetonitrile | 10000.0 | 18666.666666666697 | 168333.333333333 | 344166.6666666669 |

## Slide 6
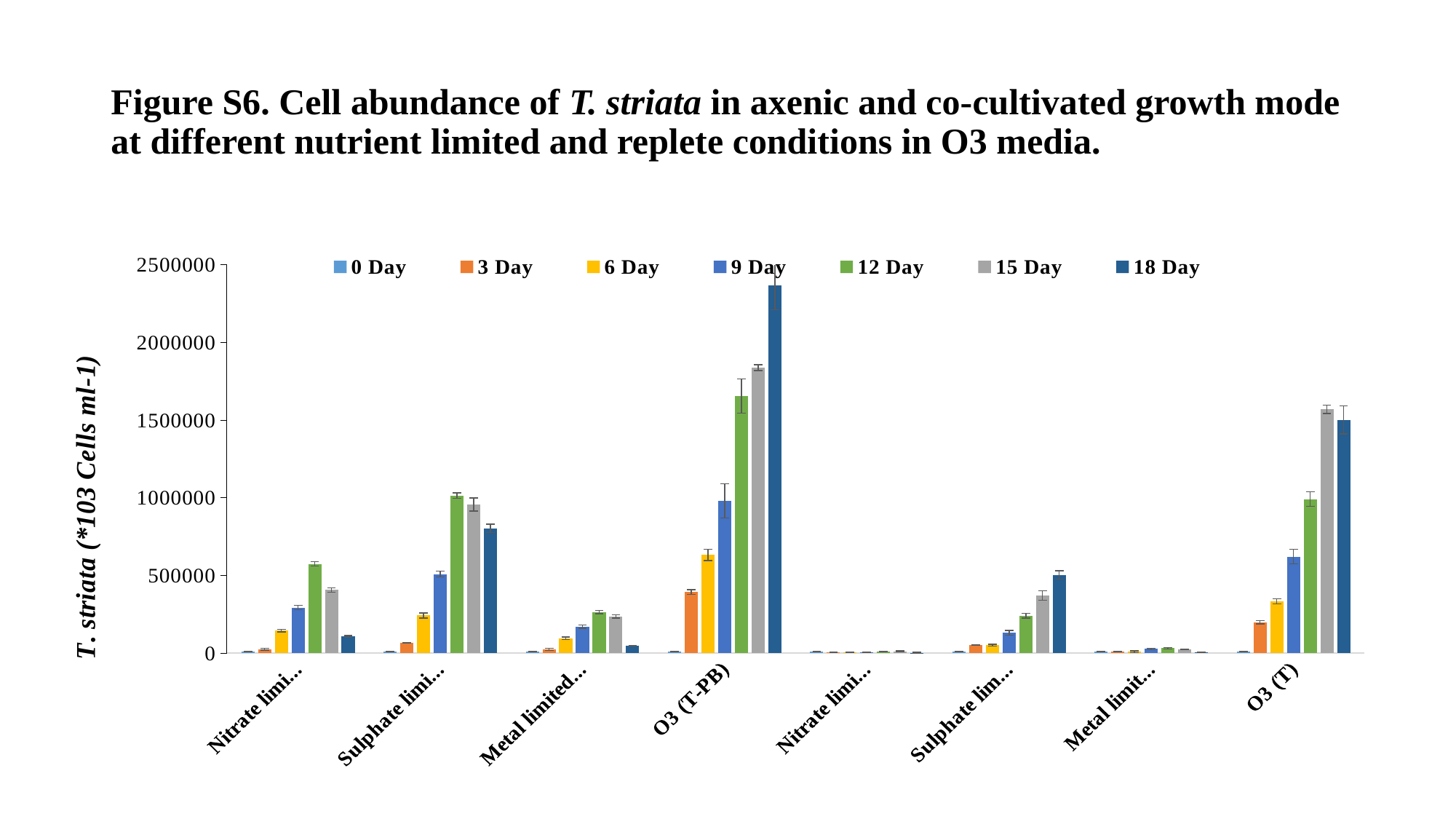

# Figure S6. Cell abundance of T. striata in axenic and co-cultivated growth mode at different nutrient limited and replete conditions in O3 media.
### Chart
| Category | 0 Day | 3 Day | 6 Day | 9 Day | 12 Day | 15 Day | 18 Day |
|---|---|---|---|---|---|---|---|
| Nitrate limited (T-PB) | 10000.0 | 25833.33333333333 | 145000.0 | 293333.3333333334 | 575000.0 | 406666.6666666667 | 107500.0 |
| Sulphate limited (T-PB ) | 10000.0 | 66666.66666666667 | 242500.0 | 509166.6666666667 | 1015000.0 | 957500.0 | 804166.6666666667 |
| Metal limited (T-PB) | 10000.0 | 24166.666666666664 | 95833.33333333333 | 171666.66666666666 | 265000.0 | 236666.66666666666 | 48333.333333333336 |
| O3 (T-PB) | 10000.0 | 394166.6666666667 | 631666.6666666667 | 980000.0 | 1655000.0 | 1838333.3333333333 | 2370000.0 |
| Nitrate limited (T) | 10000.0 | 7500.0 | 7500.0 | 6666.66666666667 | 10833.333333333332 | 10833.333333333332 | 2500.0 |
| Sulphate limited (T) | 10000.0 | 51666.66666666666 | 51666.66666666666 | 130833.33333333333 | 240833.3333333333 | 371666.6666666667 | 504166.6666666667 |
| Metal limited (T) | 10000.0 | 9166.666666666664 | 9166.666666666664 | 26666.666666666664 | 31666.666666666664 | 25000.0 | 4166.66666666667 |
| O3 (T) | 10000.0 | 199166.66666666666 | 334166.6666666667 | 621666.6666666667 | 991666.6666666667 | 1570000.0 | 1501666.666666667 |
