# Supplementary material for: Pelagibaca bermudensis promotes biofuel competence of Tetraselmis striata in a broad range of abiotic stressors: dynamics of quorum-sensing precursors and strategic improvement in lipid productivity
Source: Biotechnol Biofuels. 2018 Apr 7;11:102. doi: 10.1186/s13068-018-1097-9 (PMC5889607; doi:10.1186/s13068-018-1097-9)
Supplement: Supplementary file 2 — Additional file 2: Table S1. Post hoc t analysis for comparison of means (LSD) based on one-way ANOVA results of the data shown in Figs. 2 and 4 (Biomass productivity and total lipid content under different environmental variables and two-stage cultivation); (Table A–H). [file 13068_2018_1097_MOESM2_ESM.docx]

**Table S1. *Post hoc* analysis for comparison of means (LSD) based on oneway ANOVA results of the data shown in the Figure 1 and Fig. 5 (Biomass productivity and total lipid under different environmental variables and two stage cultivation.)**

1. **pH – Biomass productivity**

| **(I) VAR00002** | **(J) VAR00002** | **Mean Difference (I-J)** | **Std. Error** | **Sig.** | **95% Confidence Interval** | |
| --- | --- | --- | --- | --- | --- | --- |
|  |  |  |  |  | **Lower Bound** | **Upper Bound** |
| **6 T-PB** | **6 T** | **5.56000*** | **2.3585** | **0.036** | **0.4213** | **10.6987** |
|  | **8 T-PB** | **-27.04000*** | **2.3585** | **0** | **-32.1787** | **-21.9013** |
|  | **8T** | **-19.99767*** | **2.3585** | **0** | **-25.1364** | **-14.8589** |
|  | **10 T-PB** | **-21.44667*** | **2.3585** | **0** | **-26.5854** | **-16.3079** |
|  | **10T** | **-14.44467*** | **2.3585** | **0** | **-19.5834** | **-9.3059** |
| **6 T** | **6 T-PB** | **-5.56000*** | **2.3585** | **0.036** | **-10.6987** | **-0.4213** |
|  | **8 T-PB** | **-32.60000*** | **2.3585** | **0** | **-37.7387** | **-27.4613** |
|  | **8T** | **-25.55767*** | **2.3585** | **0** | **-30.6964** | **-20.4189** |
|  | **10 T-PB** | **-230 g/L T667*** | **2.3585** | **0** | **-32.1454** | **-21.8679** |
|  | **10T** | **-20.00467*** | **2.3585** | **0** | **-25.1434** | **-14.8659** |
| **8 T-PB** | **6 T-PB** | **27.04000*** | **2.3585** | **0** | **21.9013** | **32.1787** |
|  | **6T** | **32.60000*** | **2.3585** | **0** | **27.4613** | **37.7387** |
|  | **8T** | **7.04233*** | **2.3585** | **0.011** | **1.9036** | **12.1811** |
|  | **10 T-PB** | **5.59333*** | **2.3585** | **0.035** | **0.4546** | **10.7321** |
|  | **10T** | **12.59533*** | **2.3585** | **0** | **7.4566** | **17.7341** |
| **8 T** | **6 T-PB** | **19.99767*** | **2.3585** | **0** | **14.8589** | **25.1364** |
|  | **6T** | **25.55767*** | **2.3585** | **0** | **20.4189** | **30.6964** |
|  | **8 T-PB** | **-7.04233*** | **2.3585** | **0.011** | **-12.1811** | **-1.9036** |
|  | **10 T-PB** | **-1.449** | **2.3585** | **0.55** | **-6.5877** | **3.6897** |
|  | **10 T** | **5.55300*** | **2.3585** | **0.036** | **0.4143** | **10.6917** |
| **10 T-PB** | **6 T-PB** | **21.44667*** | **2.3585** | **0** | **16.3079** | **26.5854** |
|  | **6 T** | **230 g/L T667*** | **2.3585** | **0** | **21.8679** | **32.1454** |
|  | **8 T-PB** | **-5.59333*** | **2.3585** | **0.035** | **-10.7321** | **-0.4546** |
|  | **8 T** | **1.449** | **2.3585** | **0.55** | **-3.6897** | **6.5877** |
|  | **10 T** | **30 g/L T200*** | **2.3585** | **0.012** | **1.8633** | **12.1407** |
| **10 T** | **6 T-PB** | **14.44467*** | **2.3585** | **0** | **9.3059** | **19.5834** |
|  | **6 T** | **20.00467*** | **2.3585** | **0** | **14.8659** | **25.1434** |
|  | **8 T-PB** | **-12.59533*** | **2.3585** | **0** | **-17.7341** | **-7.4566** |
|  | **8 T** | **-5.55300*** | **2.3585** | **0.036** | **-10.6917** | **-0.4143** |
|  | **10 T-PB** | **-30 g/L T200*** | **2.3585** | **0.012** | **-12.1407** | **-1.8633** |
| *** The mean difference is significant at the 0.05 level.** | | | |  |  |  |

1. **pH- Lipid**

| **(I) VAR00002** | **(J) VAR00002** | **Mean Difference (I-J)** | **Std. Error** | **Sig.** | **95% Confidence Interval** | |
| --- | --- | --- | --- | --- | --- | --- |
|  |  |  |  |  | **Lower Bound** | **Upper Bound** |
| **6 T-PB** | **6T** | **1.29333*** | **0.39286** | **0.006** | **0.4374** | **2.1493** |
|  | **8 T-PB** | **2.58333*** | **0.39286** | **0** | **1.7274** | **3.4393** |
|  | **8 T** | **1.54533*** | **0.39286** | **0.002** | **0.6894** | **2.4013** |
|  | **10 T-PB** | **3.78000*** | **0.39286** | **0** | **2.924** | **4.636** |
|  | **10 T** | **0.6** | **0.39286** | **0.153** | **-0.256** | **1.456** |
| **6 T** | **6 T-PB** | **-1.29333*** | **0.39286** | **0.006** | **-2.1493** | **-0.4374** |
|  | **8 T-PB** | **1.29000*** | **0.39286** | **0.007** | **0.434** | **2.146** |
|  | **8 T** | **0.252** | **0.39286** | **0.533** | **-0.604** | **1.108** |
|  | **10 T-PB** | **2.48667*** | **0.39286** | **0** | **1.6307** | **3.3426** |
|  | **10 T** | **-0.69333** | **0.39286** | **0.103** | **-1.5493** | **0.1626** |
| **8 T-PB** | **6 T-PB** | **-2.58333*** | **0.39286** | **0** | **-3.4393** | **-1.7274** |
|  | **6 T** | **-1.29000*** | **0.39286** | **0.007** | **-2.146** | **-0.434** |
|  | **8 T** | **-1.03800*** | **0.39286** | **0.021** | **-1.894** | **-0.182** |
|  | **10 T-PB** | **1.19667*** | **0.39286** | **0.01** | **0.3407** | **2.0526** |
|  | **10 T** | **-1.98333*** | **0.39286** | **0** | **-2.8393** | **-1.1274** |
| **8 T** | **6 T-PB** | **-1.54533*** | **0.39286** | **0.002** | **-2.4013** | **-0.6894** |
|  | **6 T** | **-0.252** | **0.39286** | **0.533** | **-1.108** | **0.604** |
|  | **8 T-PB** | **1.03800*** | **0.39286** | **0.021** | **0.182** | **1.894** |
|  | **10 T-PB** | **2.23467*** | **0.39286** | **0** | **1.3787** | **3.0906** |
|  | **10 T** | **-.94533*** | **0.39286** | **0.033** | **-1.8013** | **-0.0894** |
| **10 T-PB** | **6 T-PB** | **-3.78000*** | **0.39286** | **0** | **-4.636** | **-2.924** |
|  | **6 T** | **-2.48667*** | **0.39286** | **0** | **-3.3426** | **-1.6307** |
|  | **8 T-PB** | **-1.19667*** | **0.39286** | **0.01** | **-2.0526** | **-0.3407** |
|  | **8 T** | **-2.23467*** | **0.39286** | **0** | **-3.0906** | **-1.3787** |
|  | **10 T** | **-3.18000*** | **0.39286** | **0** | **-4.036** | **-2.324** |
| **10 T** | **6 T-PB** | **-0.6** | **0.39286** | **0.153** | **-1.456** | **0.256** |
|  | **6 T** | **0.69333** | **0.39286** | **0.103** | **-0.1626** | **1.5493** |
|  | **8 T-PB** | **1.98333*** | **0.39286** | **0** | **1.1274** | **2.8393** |
|  | **8 T** | **.94533*** | **0.39286** | **0.033** | **0.0894** | **1.8013** |
|  | **10 T-PB** | **3.18000*** | **0.39286** | **0** | **2.324** | **4.036** |
| *** The mean difference is significant at the 0.05 level.** | | | | | |  |

1. **Salinity- Biomass Producitivty**

| **Dependent Variable:** | **VAR00004** |  |  |  |  |  |
| --- | --- | --- | --- | --- | --- | --- |
| **LSD** |  |  |  |  |  |  |
| **(I) VAR00002** | **Mean Difference (I-J)** | | **Std. Error** | **Sig.** | **95% Confidence Interval** | |
|  |  |  |  |  | **Lower Bound** | **Upper Bound** |
| **20 g/L T-PB** | **25 g/L T-PB** | **16.94600^*^** | **1.49924** | **.000** | **13.7677** | **20.1243** |
|  | **30 g/L T-PB** | **20.05267^*^** | **1.49924** | **.000** | **16.8744** | **23.2309** |
|  | **35 g/L T-PB** | **23.05200^*^** | **1.49924** | **.000** | **19.8737** | **26.2303** |
|  | **20 g/L T** | **7.03767^*^** | **1.49924** | **.000** | **3.8594** | **10.2159** |
|  | **25 g/L T** | **27.61833^*^** | **1.49924** | **.000** | **24.4401** | **30.7966** |
|  | **30 g/L T** | **31.16500^*^** | **1.49924** | **.000** | **27.9867** | **34.3433** |
|  | **35 g/L T** | **34.20533^*^** | **1.49924** | **.000** | **31.0271** | **37.3836** |
| **25 g/L T-PB** | **20 g/L T-PB** | **-16.94600^*^** | **1.49924** | **.000** | **-20.1243** | **-13.7677** |
|  | **30 g/L T-PB** | **3.10667** | **1.49924** | **.055** | **-.0716** | **6.2849** |
|  | **35 g/L T-PB** | **6.10600^*^** | **1.49924** | **.001** | **2.9277** | **9.2843** |
|  | **20 g/L T** | **-9.90833^*^** | **1.49924** | **.000** | **-13.0866** | **-6.7301** |
|  | **25 g/L T** | **10.67233^*^** | **1.49924** | **.000** | **7.4941** | **13.8506** |
|  | **30 g/L T** | **14.21900^*^** | **1.49924** | **.000** | **11.0407** | **17.3973** |
|  | **35 g/L T** | **17.25933^*^** | **1.49924** | **.000** | **14.0811** | **20.4376** |
| **30 g/L T-PB** | **20 g/L T-PB** | **-20.05267^*^** | **1.49924** | **.000** | **-23.2309** | **-16.8744** |
|  | **25 g/L T-PB** | **-3.10667** | **1.49924** | **.055** | **-6.2849** | **.0716** |
|  | **35 g/L T-PB** | **2.99933** | **1.49924** | **.063** | **-.1789** | **6.1776** |
|  | **20 g/L T** | **-13.01500^*^** | **1.49924** | **.000** | **-16.1933** | **-9.8367** |
|  | **25 g/L T** | **7.56567^*^** | **1.49924** | **.000** | **4.3874** | **10.7439** |
|  | **30 g/L T** | **11.11233^*^** | **1.49924** | **.000** | **7.9341** | **14.2906** |
|  | **35 g/L T** | **14.15267^*^** | **1.49924** | **.000** | **10.9744** | **17.3309** |
| **35 g/L T-PB** | **20 g/L T-PB** | **-23.05200^*^** | **1.49924** | **.000** | **-26.2303** | **-19.8737** |
|  | **25 g/L T-PB** | **-6.10600^*^** | **1.49924** | **.001** | **-9.2843** | **-2.9277** |
|  | **30 g/L T-PB** | **-2.99933** | **1.49924** | **.063** | **-6.1776** | **.1789** |
|  | **20 g/L T** | **-16.01433^*^** | **1.49924** | **.000** | **-19.1926** | **-12.8361** |
|  | **25 g/L T** | **4.56633^*^** | **1.49924** | **.008** | **1.3881** | **7.7446** |
|  | **30 g/L T** | **8.11300^*^** | **1.49924** | **.000** | **4.9347** | **11.2913** |
|  | **35 g/L T** | **11.15333^*^** | **1.49924** | **.000** | **7.9751** | **14.3316** |
| **20 g/L T** | **20 g/L T-PB** | **-7.03767^*^** | **1.49924** | **.000** | **-10.2159** | **-3.8594** |
|  | **25 g/L T-PB** | **9.90833^*^** | **1.49924** | **.000** | **6.7301** | **13.0866** |
|  | **30 g/L T-PB** | **13.01500^*^** | **1.49924** | **.000** | **9.8367** | **16.1933** |
|  | **35 g/L T-PB** | **16.01433^*^** | **1.49924** | **.000** | **12.8361** | **19.1926** |
|  | **25 g/L T** | **20.58067^*^** | **1.49924** | **.000** | **17.4024** | **23.7589** |
|  | **30 g/L T** | **24.12733^*^** | **1.49924** | **.000** | **20.9491** | **27.3056** |
|  | **35 g/L T** | **27.16767^*^** | **1.49924** | **.000** | **23.9894** | **30.3459** |
| **25 g/L T** | **20 g/L T-PB** | **-27.61833^*^** | **1.49924** | **.000** | **-30.7966** | **-24.4401** |
|  | **25 g/L T-PB** | **-10.67233^*^** | **1.49924** | **.000** | **-13.8506** | **-7.4941** |
|  | **30 g/L T-PB** | **-7.56567^*^** | **1.49924** | **.000** | **-10.7439** | **-4.3874** |
|  | **35 g/L T-PB** | **-4.56633^*^** | **1.49924** | **.008** | **-7.7446** | **-1.3881** |
|  | **20 g/L T** | **-20.58067^*^** | **1.49924** | **.000** | **-23.7589** | **-17.4024** |
|  | **7** | **3.54667^*^** | **1.49924** | **.031** | **.3684** | **6.7249** |
|  | **35 g/L T** | **6.58700^*^** | **1.49924** | **.000** | **3.4087** | **9.7653** |
| **30 g/L T** | **20 g/L T-PB** | **-31.16500^*^** | **1.49924** | **.000** | **-34.3433** | **-27.9867** |
|  | **25 g/L T-PB** | **-14.21900^*^** | **1.49924** | **.000** | **-17.3973** | **-11.0407** |
|  | **30 g/L T-PB** | **-11.11233^*^** | **1.49924** | **.000** | **-14.2906** | **-7.9341** |
|  | **35 g/L T-PB** | **-8.11300^*^** | **1.49924** | **.000** | **-11.2913** | **-4.9347** |
|  | **20 g/L T** | **-24.12733^*^** | **1.49924** | **.000** | **-27.3056** | **-20.9491** |
|  | **25 g/L T** | **-3.54667^*^** | **1.49924** | **.031** | **-6.7249** | **-.3684** |
|  | **35 g/L T** | **3.04033** | **1.49924** | **.060** | **-.1379** | **6.2186** |
| **35 g/L T** | **20 g/L T-PB** | **-34.20533^*^** | **1.49924** | **.000** | **-37.3836** | **-31.0271** |
|  | **25 g/L T-PB** | **-17.25933^*^** | **1.49924** | **.000** | **-20.4376** | **-14.0811** |
|  | **30 g/L T-PB** | **-14.15267^*^** | **1.49924** | **.000** | **-17.3309** | **-10.9744** |
|  | **35 g/L T-PB** | **-11.15333^*^** | **1.49924** | **.000** | **-14.3316** | **-7.9751** |
|  | **20 g/L T** | **-27.16767^*^** | **1.49924** | **.000** | **-30.3459** | **-23.9894** |
|  | **25 g/L T** | **-6.58700^*^** | **1.49924** | **.000** | **-9.7653** | **-3.4087** |
|  | **30 g/L T** | **-3.04033** | **1.49924** | **.060** | **-6.2186** | **.1379** |
| ***. The mean difference is significant at the 0.05 level.** | | | | | | |

**D. Salinity - Lipid**

| **(I) VAR00002** | | **Mean Difference (I-J)** | **Std. Error** | **Sig.** | **95% Confidence Interval** | |
| --- | --- | --- | --- | --- | --- | --- |
|  |  |  |  |  | **Lower Bound** | **Upper Bound** |
| **20 g/L T-PB** | **25 g/L T-PB** | **-2.24767^*^** | **1.01242** | **.041** | **-4.3939** | **-.1014** |
|  | **30 g/L T-PB** | **1.54467** | **1.01242** | **.147** | **-.6016** | **3.6909** |
|  | **35 g/L T-PB** | **1.89200** | **1.01242** | **.080** | **-.2542** | **4.0382** |
|  | **20 g/L T** | **.83667** | **1.01242** | **.421** | **-1.3096** | **2.9829** |
|  | **25 g/L T** | **-1.62333** | **1.01242** | **.128** | **-3.7696** | **.5229** |
|  | **30 g/L T** | **-2.31333^*^** | **1.01242** | **.036** | **-4.4596** | **-.1671** |
|  | **35 g/L T** | **-1.08667** | **1.01242** | **.299** | **-3.2329** | **1.0596** |
| **25 g/L T-PB** | **20 g/L T-PB** | **2.24767^*^** | **1.01242** | **.041** | **.1014** | **4.3939** |
|  | **30 g/L T-PB** | **3.79233^*^** | **1.01242** | **.002** | **1.6461** | **5.9386** |
|  | **35 g/L T-PB** | **4.13967^*^** | **1.01242** | **.001** | **1.9934** | **6.2859** |
|  | **20 g/L T** | **3.08433^*^** | **1.01242** | **.008** | **.9381** | **5.2306** |
|  | **25 g/L T** | **.62433** | **1.01242** | **.546** | **-1.5219** | **2.7706** |
|  | **30 g/L T** | **-.06567** | **1.01242** | **.949** | **-2.2119** | **2.0806** |
|  | **35 g/L T** | **1.16100** | **1.01242** | **.268** | **-.9852** | **3.3072** |
| **30 g/L T-PB** | **20 g/L T-PB** | **-1.54467** | **1.01242** | **.147** | **-3.6909** | **.6016** |
|  | **25 g/L T-PB** | **-3.79233^*^** | **1.01242** | **.002** | **-5.9386** | **-1.6461** |
|  | **35 g/L T-PB** | **.34733** | **1.01242** | **.736** | **-1.7989** | **2.4936** |
|  | **20 g/L T** | **-.70800** | **1.01242** | **.494** | **-2.8542** | **1.4382** |
|  | **25 g/L T** | **-3.16800^*^** | **1.01242** | **.006** | **-5.3142** | **-1.0218** |
|  | **30 g/L T** | **-3.85800^*^** | **1.01242** | **.002** | **-6.0042** | **-1.7118** |
|  | **35 g/L T** | **-2.63133^*^** | **1.01242** | **.019** | **-4.7776** | **-.4851** |
| **35 g/L T-PB** | **20 g/L T-PB** | **-1.89200** | **1.01242** | **.080** | **-4.0382** | **.2542** |
|  | **25 g/L T-PB** | **-4.13967^*^** | **1.01242** | **.001** | **-6.2859** | **-1.9934** |
|  | **30 g/L T-PB** | **-.34733** | **1.01242** | **.736** | **-2.4936** | **1.7989** |
|  | **20 g/L T** | **-1.05533** | **1.01242** | **.313** | **-3.2016** | **1.0909** |
|  | **25 g/L T** | **-3.51533^*^** | **1.01242** | **.003** | **-5.6616** | **-1.3691** |
|  | **30 g/L T** | **-4.20533^*^** | **1.01242** | **.001** | **-6.3516** | **-2.0591** |
|  | **35 g/L T** | **-2.97867^*^** | **1.01242** | **.010** | **-5.1249** | **-.8324** |
| **20 g/L T** | **20 g/L T-PB** | **-.83667** | **1.01242** | **.421** | **-2.9829** | **1.3096** |
|  | **25 g/L T-PB** | **-3.08433^*^** | **1.01242** | **.008** | **-5.2306** | **-.9381** |
|  | **30 g/L T-PB** | **.70800** | **1.01242** | **.494** | **-1.4382** | **2.8542** |
|  | **35 g/L T-PB** | **1.05533** | **1.01242** | **.313** | **-1.0909** | **3.2016** |
|  | **25 g/L T** | **-2.46000^*^** | **1.01242** | **.027** | **-4.6062** | **-.3138** |
|  | **30 g/L T** | **-3.15000^*^** | **1.01242** | **.007** | **-5.2962** | **-1.0038** |
|  | **35 g/L T** | **-1.92333** | **1.01242** | **.076** | **-4.0696** | **.2229** |
| **25 g/L T** | **20 g/L T-PB** | **1.62333** | **1.01242** | **.128** | **-.5229** | **3.7696** |
|  | **25 g/L T-PB** | **-.62433** | **1.01242** | **.546** | **-2.7706** | **1.5219** |
|  | **30 g/L T-PB** | **3.16800^*^** | **1.01242** | **.006** | **1.0218** | **5.3142** |
|  | **35 g/L T-PB** | **3.51533^*^** | **1.01242** | **.003** | **1.3691** | **5.6616** |
|  | **20 g/L T** | **2.46000^*^** | **1.01242** | **.027** | **.3138** | **4.6062** |
|  | **30 g/L T** | **-.69000** | **1.01242** | **.505** | **-2.8362** | **1.4562** |
|  | **35 g/L T** | **.53667** | **1.01242** | **.603** | **-1.6096** | **2.6829** |
| **30 g/L T** | **20 g/L T-PB** | **2.31333^*^** | **1.01242** | **.036** | **.1671** | **4.4596** |
|  | **25 g/L T-PB** | **.06567** | **1.01242** | **.949** | **-2.0806** | **2.2119** |
|  | **30 g/L T-PB** | **3.85800^*^** | **1.01242** | **.002** | **1.7118** | **6.0042** |
|  | **35 g/L T-PB** | **4.20533^*^** | **1.01242** | **.001** | **2.0591** | **6.3516** |
|  | **20 g/L T** | **3.15000^*^** | **1.01242** | **.007** | **1.0038** | **5.2962** |
|  | **25 g/L T** | **.69000** | **1.01242** | **.505** | **-1.4562** | **2.8362** |
|  | **35 g/L T** | **1.22667** | **1.01242** | **.243** | **-.9196** | **3.3729** |
| **35 g/L T** | **20 g/L T-PB** | **1.08667** | **1.01242** | **.299** | **-1.0596** | **3.2329** |
|  | **25 g/L T-PB** | **-1.16100** | **1.01242** | **.268** | **-3.3072** | **.9852** |
|  | **30 g/L T-PB** | **2.63133^*^** | **1.01242** | **.019** | **.4851** | **4.7776** |
|  | **35 g/L T-PB** | **2.97867^*^** | **1.01242** | **.010** | **.8324** | **5.1249** |
|  | **20 g/L T** | **1.92333** | **1.01242** | **.076** | **-.2229** | **4.0696** |
|  | **25 g/L T** | **-.53667** | **1.01242** | **.603** | **-2.6829** | **1.6096** |
|  | **30 g/L T** | **-1.22667** | **1.01242** | **.243** | **-3.3729** | **.9196** |
| ***. The mean difference is significant at the 0.05 level.** | | | | | | |

**E) Light and Temperature –Biomass Productivity**

| **(I) VAR00002** | **(J) VAR00002** | **Mean Difference (I-J)** | **Std. Error** | **Sig.** | **95% Confidence Interval** | |
| --- | --- | --- | --- | --- | --- | --- |
|  |  |  |  |  | **Lower Bound** | **Upper Bound** |
| **T-PB (Low Light)** | **T-PB (Low Temp.)** | **-5.03704** | **4.53004** | **0.283** | **-14.6403** | **4.5662** |
|  | **T-PB (High Light)** | **13.48148*** | **4.53004** | **0.009** | **3.8782** | **23.0847** |
|  | **T-PB (Normal Light and Temp)** | **-17.62963*** | **4.53004** | **0.001** | **-27.2329** | **-8.0264** |
|  | **T (Low Light)** | **8.88889** | **4.53004** | **0.067** | **-0.7144** | **18.4921** |
|  | **T (Low Temp.)** | **10.81481*** | **4.53004** | **0.03** | **1.2116** | **20.4181** |
|  | **T (High Light)** | **19.85185*** | **4.53004** | **0** | **10.2486** | **29.4551** |
|  | **T (Normal Light and Temp)** | **-41.48148*** | **4.53004** | **0** | **-51.0847** | **-31.8782** |
| **T-PB (Low Temp.)** | **T-PB (Low Light)** | **5.03704** | **4.53004** | **0.283** | **-4.5662** | **14.6403** |
|  | **T-PB (High Light)** | **18.51852*** | **4.53004** | **0.001** | **8.9153** | **28.1218** |
|  | **T-PB (Normal Light and Temp)** | **-12.59259*** | **4.53004** | **0.013** | **-22.1958** | **-2.9893** |
|  | **T (Low Light)** | **13.92593*** | **4.53004** | **0.007** | **4.3227** | **23.5292** |
|  | **T (Low Temp.)** | **15.85185*** | **4.53004** | **0.003** | **6.2486** | **25.4551** |
|  | **T (High Light)** | **24.88889*** | **4.53004** | **0** | **15.2856** | **34.4921** |
|  | **T (Normal Light and Temp)** | **-36.44444*** | **4.53004** | **0** | **-46.0477** | **-26.8412** |
| **T-PB (High Light)** | **T-PB (Low Light)** | **-13.48148*** | **4.53004** | **0.009** | **-23.0847** | **-3.8782** |
|  | **T-PB (Low Temp.)** | **-18.51852*** | **4.53004** | **0.001** | **-28.1218** | **-8.9153** |
|  | **T-PB (Normal Light and Temp)** | **-31.11111*** | **4.53004** | **0** | **-40.7144** | **-21.5079** |
|  | **T (Low Light)** | **-4.59259** | **4.53004** | **0.326** | **-14.1958** | **5.0107** |
|  | **T (Low Temp.)** | **-2.66667** | **4.53004** | **0.564** | **-12.2699** | **6.9366** |
|  | **T (High Light)** | **6.37037** | **4.53004** | **0.179** | **-3.2329** | **15.9736** |
|  | **T (Normal Light and Temp)** | **-54.96296*** | **4.53004** | **0** | **-64.5662** | **-45.3597** |
| **T-PB (Normal Light and Temp)** | **T-PB (Low Light)** | **17.62963*** | **4.53004** | **0.001** | **8.0264** | **27.2329** |
|  | **T-PB (Low Temp.)** | **12.59259*** | **4.53004** | **0.013** | **2.9893** | **22.1958** |
|  | **T-PB (High Light)** | **31.11111*** | **4.53004** | **0** | **21.5079** | **40.7144** |
|  | **T (Low Light)** | **26.51852*** | **4.53004** | **0** | **16.9153** | **36.1218** |
|  | **T (Low Temp.)** | **28.44444*** | **4.53004** | **0** | **18.8412** | **38.0477** |
|  | **T (High Light)** | **37.48148*** | **4.53004** | **0** | **27.8782** | **47.0847** |
|  | **T (Normal Light and Temp)** | **-23.85185*** | **4.53004** | **0** | **-33.4551** | **-14.2486** |
| **T (Low Light)** | **T-PB (Low Light)** | **-8.88889** | **4.53004** | **0.067** | **-18.4921** | **0.7144** |
|  | **T-PB (Low Temp.)** | **-13.92593*** | **4.53004** | **0.007** | **-23.5292** | **-4.3227** |
|  | **T-PB (High Light)** | **4.59259** | **4.53004** | **0.326** | **-5.0107** | **14.1958** |
|  | **T-PB (Normal Light and Temp)** | **-26.51852*** | **4.53004** | **0** | **-36.1218** | **-16.9153** |
|  | **T (Low Temp.)** | **1.92593** | **4.53004** | **0.676** | **-7.6773** | **11.5292** |
|  | **T (High Light)** | **10.96296*** | **4.53004** | **0.028** | **1.3597** | **20.5662** |
|  | **T (Normal Light and Temp)** | **-50.37037*** | **4.53004** | **0** | **-59.9736** | **-40.7671** |
| **T (Low Temp.)** | **T-PB (Low Light)** | **-10.81481*** | **4.53004** | **0.03** | **-20.4181** | **-1.2116** |
|  | **T-PB (Low Temp.)** | **-15.85185*** | **4.53004** | **0.003** | **-25.4551** | **-6.2486** |
|  | **T-PB (High Light)** | **2.66667** | **4.53004** | **0.564** | **-6.9366** | **12.2699** |
|  | **T-PB (Normal Light and Temp)** | **-28.44444*** | **4.53004** | **0** | **-38.0477** | **-18.8412** |
|  | **T (Low Light)** | **-1.92593** | **4.53004** | **0.676** | **-11.5292** | **7.6773** |
|  | **T (High Light)** | **9.03704** | **4.53004** | **0.063** | **-0.5662** | **18.6403** |
|  | **T (Normal Light and Temp)** | **-52.29630*** | **4.53004** | **0** | **-61.8996** | **-42.693** |
| **T (High Light)** | **T-PB (Low Light)** | **-19.85185*** | **4.53004** | **0** | **-29.4551** | **-10.2486** |
|  | **T-PB (Low Temp.)** | **-24.88889*** | **4.53004** | **0** | **-34.4921** | **-15.2856** |
|  | **T-PB (High Light)** | **-6.37037** | **4.53004** | **0.179** | **-15.9736** | **3.2329** |
|  | **T-PB (Normal Light and Temp)** | **-37.48148*** | **4.53004** | **0** | **-47.0847** | **-27.8782** |
|  | **T (Low Light)** | **-10.96296*** | **4.53004** | **0.028** | **-20.5662** | **-1.3597** |
|  | **T (Low Temp.)** | **-9.03704** | **4.53004** | **0.063** | **-18.6403** | **0.5662** |
|  | **T (Normal Light and Temp)** | **-61.33333*** | **4.53004** | **0** | **-70.9366** | **-51.7301** |
| **T (Normal Light and Temp)** | **T-PB (Low Light)** | **41.48148*** | **4.53004** | **0** | **31.8782** | **51.0847** |
|  | **T-PB (Low Temp.)** | **36.44444*** | **4.53004** | **0** | **26.8412** | **46.0477** |
|  | **T-PB (High Light)** | **54.96296*** | **4.53004** | **0** | **45.3597** | **64.5662** |
|  | **T-PB (Normal Light and Temp)** | **23.85185*** | **4.53004** | **0** | **14.2486** | **33.4551** |
|  | **T (Low Light)** | **50.37037*** | **4.53004** | **0** | **40.7671** | **59.9736** |
|  | **T (Low Temp.)** | **52.29630*** | **4.53004** | **0** | **42.693** | **61.8996** |
|  | **T (High Light)** | **61.33333*** | **4.53004** | **0** | **51.7301** | **70.9366** |
| *** The mean difference is significant at the 0.05 level.** | | | | | |  |

**F) Light and Temperature - Lipid**

| **LSD** |  |  |  |  |  |  |
| --- | --- | --- | --- | --- | --- | --- |
|  |  |  |  |  |  |  |
| **(I) VAR00002** | **(J) VAR00002** | **Mean Difference (I-J)** | **Std. Error** | **Sig.** | **95% Confidence Interval** | |
|  |  |  |  |  | **Lower Bound** | **Upper Bound** |
| **T-PB (Low Light)** | **T-PB (Low Temp.)** | **-6.10145*** | **1.40264** | **0** | **-9.0749** | **-3.128** |
|  | **T-PB (High Light)** | **1.18138** | **1.40264** | **0.412** | **-1.7921** | **4.1548** |
|  | **T-PB (Normal Light and Temp)** | **-1.66478** | **1.40264** | **0.253** | **-4.6382** | **1.3087** |
|  | **T (Low Light)** | **-2.89855** | **1.40264** | **0.055** | **-5.872** | **0.0749** |
|  | **T (Low Temp.)** | **-8.69565*** | **1.40264** | **0** | **-11.6691** | **-5.7222** |
|  | **T (High Light)** | **-0.72464** | **1.40264** | **0.612** | **-3.6981** | **2.2488** |
|  | **T (Normal Light and Temp)** | **-2.657** | **1.40264** | **0.076** | **-5.6305** | **0.3165** |
| **T-PB (Low Temp.)** | **T-PB (Low Light)** | **6.10145*** | **1.40264** | **0** | **3.128** | **9.0749** |
|  | **T-PB (High Light)** | **7.28283*** | **1.40264** | **0** | **4.3094** | **10.2563** |
|  | **T-PB (Normal Light and Temp)** | **4.43667*** | **1.40264** | **0.006** | **1.4632** | **7.4101** |
|  | **T (Low Light)** | **3.20290*** | **1.40264** | **0.036** | **0.2294** | **6.1764** |
|  | **T (Low Temp.)** | **-2.5942** | **1.40264** | **0.083** | **-5.5677** | **0.3793** |
|  | **T (High Light)** | **5.37681*** | **1.40264** | **0.001** | **2.4034** | **8.3503** |
|  | **T (Normal Light and Temp)** | **3.44444*** | **1.40264** | **0.026** | **0.471** | **6.4179** |
| **T-PB (High Light)** | **T-PB (Low Light)** | **-1.18138** | **1.40264** | **0.412** | **-4.1548** | **1.7921** |
|  | **T-PB (Low Temp.)** | **-7.28283*** | **1.40264** | **0** | **-10.2563** | **-4.3094** |
|  | **T-PB (Normal Light and Temp)** | **-2.84616** | **1.40264** | **0.059** | **-5.8196** | **0.1273** |
|  | **T (Low Light)** | **-4.07993*** | **1.40264** | **0.01** | **-7.0534** | **-1.1065** |
|  | **T (Low Temp.)** | **-9.87703*** | **1.40264** | **0** | **-12.8505** | **-6.9036** |
|  | **T (High Light)** | **-1.90602** | **1.40264** | **0.193** | **-4.8795** | **1.0674** |
|  | **T (Normal Light and Temp)** | **-3.83838*** | **1.40264** | **0.015** | **-6.8118** | **-0.8649** |
| **T-PB (Normal Light and Temp)** | **T-PB (Low Light)** | **1.66478** | **1.40264** | **0.253** | **-1.3087** | **4.6382** |
|  | **T-PB (Low Temp.)** | **-4.43667*** | **1.40264** | **0.006** | **-7.4101** | **-1.4632** |
|  | **T-PB (High Light)** | **2.84616** | **1.40264** | **0.059** | **-0.1273** | **5.8196** |
|  | **T (Low Light)** | **-1.23377** | **1.40264** | **0.392** | **-4.2072** | **1.7397** |
|  | **T (Low Temp.)** | **-7.03087*** | **1.40264** | **0** | **-10.0043** | **-4.0574** |
|  | **T (High Light)** | **0.94014** | **1.40264** | **0.512** | **-2.0333** | **3.9136** |
|  | **T (Normal Light and Temp)** | **-0.99222** | **1.40264** | **0.489** | **-3.9657** | **1.9812** |
| **T (Low Light)** | **T-PB (Low Light)** | **2.89855** | **1.40264** | **0.055** | **-0.0749** | **5.872** |
|  | **T-PB (Low Temp.)** | **-3.20290*** | **1.40264** | **0.036** | **-6.1764** | **-0.2294** |
|  | **T-PB (High Light)** | **4.07993*** | **1.40264** | **0.01** | **1.1065** | **7.0534** |
|  | **T-PB (Normal Light and Temp)** | **1.23377** | **1.40264** | **0.392** | **-1.7397** | **4.2072** |
|  | **T (Low Temp.)** | **-5.79710*** | **1.40264** | **0.001** | **-8.7706** | **-2.8236** |
|  | **T (High Light)** | **2.17391** | **1.40264** | **0.141** | **-0.7995** | **5.1474** |
|  | **T (Normal Light and Temp)** | **0.24155** | **1.40264** | **0.865** | **-2.7319** | **3.215** |
| **T (Low Temp.)** | **T-PB (Low Light)** | **8.69565*** | **1.40264** | **0** | **5.7222** | **11.6691** |
|  | **T-PB (Low Temp.)** | **2.5942** | **1.40264** | **0.083** | **-0.3793** | **5.5677** |
|  | **T-PB (High Light)** | **9.87703*** | **1.40264** | **0** | **6.9036** | **12.8505** |
|  | **T-PB (Normal Light and Temp)** | **7.03087*** | **1.40264** | **0** | **4.0574** | **10.0043** |
|  | **T (Low Light)** | **5.79710*** | **1.40264** | **0.001** | **2.8236** | **8.7706** |
|  | **T (High Light)** | **7.97101*** | **1.40264** | **0** | **4.9976** | **10.9445** |
|  | **T (Normal Light and Temp)** | **6.03865*** | **1.40264** | **0.001** | **3.0652** | **9.0121** |
| **T (High Light)** | **T-PB (Low Light)** | **0.72464** | **1.40264** | **0.612** | **-2.2488** | **3.6981** |
|  | **T-PB (Low Temp.)** | **-5.37681*** | **1.40264** | **0.001** | **-8.3503** | **-2.4034** |
|  | **T-PB (High Light)** | **1.90602** | **1.40264** | **0.193** | **-1.0674** | **4.8795** |
|  | **T-PB (Normal Light and Temp)** | **-0.94014** | **1.40264** | **0.512** | **-3.9136** | **2.0333** |
|  | **T (Low Light)** | **-2.17391** | **1.40264** | **0.141** | **-5.1474** | **0.7995** |
|  | **T (Low Temp.)** | **-7.97101*** | **1.40264** | **0** | **-10.9445** | **-4.9976** |
|  | **T (Normal Light and Temp)** | **-1.93237** | **1.40264** | **0.187** | **-4.9058** | **1.0411** |
| **T (Normal Light and Temp)** | **T-PB (Low Light)** | **2.657** | **1.40264** | **0.076** | **-0.3165** | **5.6305** |
|  | **T-PB (Low Temp.)** | **-3.44444*** | **1.40264** | **0.026** | **-6.4179** | **-0.471** |
|  | **T-PB (High Light)** | **3.83838*** | **1.40264** | **0.015** | **0.8649** | **6.8118** |
|  | **T-PB (Normal Light and Temp)** | **0.99222** | **1.40264** | **0.489** | **-1.9812** | **3.9657** |
|  | **T (Low Light)** | **-0.24155** | **1.40264** | **0.865** | **-3.215** | **2.7319** |
|  | **T (Low Temp.)** | **-6.03865*** | **1.40264** | **0.001** | **-9.0121** | **-3.0652** |
|  | **T (High Light)** | **1.93237** | **1.40264** | **0.187** | **-1.0411** | **4.9058** |
| *** The mean difference is significant at the 0.05 level.** | | | | | |  |

**G. Nutrient limitation – Biomass Productivity**

| **SPSS posthoc code** | **Exp. condition** | **Axenic/ Co-cultivated** |
| --- | --- | --- |
| **1** | **Sulphate Limitation** | **T** |
| **2** |  | **T-PB** |
| **3** | **Metal Limitation** | **T** |
| **4** |  | **T-PB** |
| **5** | **Nitrate Limitation** | **T** |
| **6** |  | **T-PB** |
| **7** | **Two stage- Salinity increase** | **T** |
| **8** |  | **T-PB** |
| **9** | **Two stage-pH decrease** | **T** |
| **10** |  | **T-PB** |
| **11** | **Two stage-Nitrate Limitation** | **T** |
| **12** |  | **T-PB** |
| **13** | **O3-Control (Nutrient Replete)** | **T** |
| **14** |  | **T-PB** |
|  |  |  |
| **Biomass Productivity** |  |  |

| **(I) VAR00002** | | **Mean Difference (I-J)** | **Std. Error** | **Sig.** | **95% Confidence Interval** | |
| --- | --- | --- | --- | --- | --- | --- |
|  |  |  |  |  | **Lower Bound** | **Upper Bound** |
| **1.00** | **2.00** | **-8.33333*** | **1.41200** | **.000** | **-11.2257** | **-5.4410** |
|  | **3.00** | **3.14815*** | **1.41200** | **.034** | **.2558** | **6.0405** |
|  | **4.00** | **-.37037** | **1.41200** | **.795** | **-3.2627** | **2.5220** |
|  | **5.00** | **8.51852*** | **1.41200** | **.000** | **5.6262** | **11.4109** |
|  | **6.00** | **-5.87963*** | **1.41200** | **.000** | **-8.7720** | **-2.9873** |
|  | **7.00** | **-4.21296*** | **1.41200** | **.006** | **-7.1053** | **-1.3206** |
|  | **8.00** | **-17.91667*** | **1.41200** | **.000** | **-20.8090** | **-15.0243** |
|  | **9.00** | **-4.62963*** | **1.41200** | **.003** | **-7.5220** | **-1.7373** |
|  | **10.00** | **-11.01852*** | **1.41200** | **.000** | **-13.9109** | **-8.1262** |
|  | **11.00** | **-19.67593*** | **1.41200** | **.000** | **-22.5683** | **-16.7836** |
|  | **12.00** | **-28.35648*** | **1.41200** | **.000** | **-31.2488** | **-25.4641** |
|  | **13.00** | **-25.22915*** | **1.41200** | **.000** | **-28.1215** | **-22.3368** |
|  | **14.00** | **-34.40581*** | **1.41200** | **.000** | **-37.2982** | **-31.5135** |
| **2.00** | **1.00** | **8.33333*** | **1.41200** | **.000** | **5.4410** | **11.2257** |
|  | **3.00** | **11.48148*** | **1.41200** | **.000** | **8.5891** | **14.3738** |
|  | **4.00** | **7.96296*** | **1.41200** | **.000** | **5.0706** | **10.8553** |
|  | **5.00** | **16.85185*** | **1.41200** | **.000** | **13.9595** | **19.7442** |
|  | **6.00** | **2.45370** | **1.41200** | **.093** | **-.4387** | **5.3461** |
|  | **7.00** | **4.12037*** | **1.41200** | **.007** | **1.2280** | **7.0127** |
|  | **8.00** | **-9.58333*** | **1.41200** | **.000** | **-12.4757** | **-6.6910** |
|  | **9.00** | **3.70370*** | **1.41200** | **.014** | **.8113** | **6.5961** |
|  | **10.00** | **-2.68519** | **1.41200** | **.068** | **-5.5775** | **.2072** |
|  | **11.00** | **-11.34259*** | **1.41200** | **.000** | **-14.2349** | **-8.4502** |
|  | **12.00** | **-20.02315*** | **1.41200** | **.000** | **-22.9155** | **-17.1308** |
|  | **13.00** | **-16.89581*** | **1.41200** | **.000** | **-19.7882** | **-14.0035** |
|  | **14.00** | **-26.07248*** | **1.41200** | **.000** | **-28.9648** | **-23.1801** |
| **3.00** | **1.00** | **-3.14815*** | **1.41200** | **.034** | **-6.0405** | **-.2558** |
|  | **2.00** | **-11.48148*** | **1.41200** | **.000** | **-14.3738** | **-8.5891** |
|  | **4.00** | **-3.51852*** | **1.41200** | **.019** | **-6.4109** | **-.6262** |
|  | **5.00** | **5.37037*** | **1.41200** | **.001** | **2.4780** | **8.2627** |
|  | **6.00** | **-9.02778*** | **1.41200** | **.000** | **-11.9201** | **-6.1354** |
|  | **7.00** | **-7.36111*** | **1.41200** | **.000** | **-10.2535** | **-4.4688** |
|  | **8.00** | **-21.06481*** | **1.41200** | **.000** | **-23.9572** | **-18.1725** |
|  | **9.00** | **-7.77778*** | **1.41200** | **.000** | **-10.6701** | **-4.8854** |
|  | **10.00** | **-14.16667*** | **1.41200** | **.000** | **-17.0590** | **-11.2743** |
|  | **11.00** | **-22.82407*** | **1.41200** | **.000** | **-25.7164** | **-19.9317** |
|  | **12.00** | **-31.50463*** | **1.41200** | **.000** | **-34.3970** | **-28.6123** |
|  | **13.00** | **-28.37730*** | **1.41200** | **.000** | **-31.2697** | **-25.4849** |
|  | **14.00** | **-37.55396*** | **1.41200** | **.000** | **-40.4463** | **-34.6616** |
| **4.00** | **1.00** | **.37037** | **1.41200** | **.795** | **-2.5220** | **3.2627** |
|  | **2.00** | **-7.96296*** | **1.41200** | **.000** | **-10.8553** | **-5.0706** |
|  | **3.00** | **3.51852*** | **1.41200** | **.019** | **.6262** | **6.4109** |
|  | **5.00** | **8.88889*** | **1.41200** | **.000** | **5.9965** | **11.7812** |
|  | **6.00** | **-5.50926*** | **1.41200** | **.001** | **-8.4016** | **-2.6169** |
|  | **7.00** | **-3.84259*** | **1.41200** | **.011** | **-6.7349** | **-.9502** |
|  | **8.00** | **-17.54630*** | **1.41200** | **.000** | **-20.4387** | **-14.6539** |
|  | **9.00** | **-4.25926*** | **1.41200** | **.005** | **-7.1516** | **-1.3669** |
|  | **10.00** | **-10.64815*** | **1.41200** | **.000** | **-13.5405** | **-7.7558** |
|  | **11.00** | **-19.30556*** | **1.41200** | **.000** | **-22.1979** | **-16.4132** |
|  | **12.00** | **-27.98611*** | **1.41200** | **.000** | **-30.8785** | **-25.0938** |
|  | **13.00** | **-24.85878*** | **1.41200** | **.000** | **-27.7511** | **-21.9664** |
|  | **14.00** | **-34.03544*** | **1.41200** | **.000** | **-36.9278** | **-31.1431** |
| **5.00** | **1.00** | **-8.51852*** | **1.41200** | **.000** | **-11.4109** | **-5.6262** |
|  | **2.00** | **-16.85185*** | **1.41200** | **.000** | **-19.7442** | **-13.9595** |
|  | **3.00** | **-5.37037*** | **1.41200** | **.001** | **-8.2627** | **-2.4780** |
|  | **4.00** | **-8.88889*** | **1.41200** | **.000** | **-11.7812** | **-5.9965** |
|  | **6.00** | **-14.39815*** | **1.41200** | **.000** | **-17.2905** | **-11.5058** |
|  | **7.00** | **-12.73148*** | **1.41200** | **.000** | **-15.6238** | **-9.8391** |
|  | **8.00** | **-26.43519*** | **1.41200** | **.000** | **-29.3275** | **-23.5428** |
|  | **9.00** | **-13.14815*** | **1.41200** | **.000** | **-16.0405** | **-10.2558** |
|  | **10.00** | **-19.53704*** | **1.41200** | **.000** | **-22.4294** | **-16.6447** |
|  | **11.00** | **-28.19444*** | **1.41200** | **.000** | **-31.0868** | **-25.3021** |
|  | **12.00** | **-36.87500*** | **1.41200** | **.000** | **-39.7674** | **-33.9826** |
|  | **13.00** | **-33.74767*** | **1.41200** | **.000** | **-36.6400** | **-30.8553** |
|  | **14.00** | **-42.92433*** | **1.41200** | **.000** | **-45.8167** | **-40.0320** |
| **6.00** | **1.00** | **5.87963*** | **1.41200** | **.000** | **2.9873** | **8.7720** |
|  | **2.00** | **-2.45370** | **1.41200** | **.093** | **-5.3461** | **.4387** |
|  | **3.00** | **9.02778*** | **1.41200** | **.000** | **6.1354** | **11.9201** |
|  | **4.00** | **5.50926*** | **1.41200** | **.001** | **2.6169** | **8.4016** |
|  | **5.00** | **14.39815*** | **1.41200** | **.000** | **11.5058** | **17.2905** |
|  | **7.00** | **1.66667** | **1.41200** | **.248** | **-1.2257** | **4.5590** |
|  | **8.00** | **-12.03704*** | **1.41200** | **.000** | **-14.9294** | **-9.1447** |
|  | **9.00** | **1.25000** | **1.41200** | **.384** | **-1.6424** | **4.1424** |
|  | **10.00** | **-5.13889*** | **1.41200** | **.001** | **-8.0312** | **-2.2465** |
|  | **11.00** | **-13.79630*** | **1.41200** | **.000** | **-16.6887** | **-10.9039** |
|  | **12.00** | **-22.47685*** | **1.41200** | **.000** | **-25.3692** | **-19.5845** |
|  | **13.00** | **-19.34952*** | **1.41200** | **.000** | **-22.2419** | **-16.4572** |
|  | **14.00** | **-28.52619*** | **1.41200** | **.000** | **-31.4185** | **-25.6338** |
| **7.00** | **1.00** | **4.21296*** | **1.41200** | **.006** | **1.3206** | **7.1053** |
|  | **2.00** | **-4.12037*** | **1.41200** | **.007** | **-7.0127** | **-1.2280** |
|  | **3.00** | **7.36111*** | **1.41200** | **.000** | **4.4688** | **10.2535** |
|  | **4.00** | **3.84259*** | **1.41200** | **.011** | **.9502** | **6.7349** |
|  | **5.00** | **12.73148*** | **1.41200** | **.000** | **9.8391** | **15.6238** |
|  | **6.00** | **-1.66667** | **1.41200** | **.248** | **-4.5590** | **1.2257** |
|  | **8.00** | **-13.70370*** | **1.41200** | **.000** | **-16.5961** | **-10.8113** |
|  | **9.00** | **-.41667** | **1.41200** | **.770** | **-3.3090** | **2.4757** |
|  | **10.00** | **-6.80556*** | **1.41200** | **.000** | **-9.6979** | **-3.9132** |
|  | **11.00** | **-15.46296*** | **1.41200** | **.000** | **-18.3553** | **-12.5706** |
|  | **12.00** | **-24.14352*** | **1.41200** | **.000** | **-27.0359** | **-21.2512** |
|  | **13.00** | **-21.01619*** | **1.41200** | **.000** | **-23.9085** | **-18.1238** |
|  | **14.00** | **-30.19285*** | **1.41200** | **.000** | **-33.0852** | **-27.3005** |
| **8.00** | **1.00** | **17.91667*** | **1.41200** | **.000** | **15.0243** | **20.8090** |
|  | **2.00** | **9.58333*** | **1.41200** | **.000** | **6.6910** | **12.4757** |
|  | **3.00** | **21.06481*** | **1.41200** | **.000** | **18.1725** | **23.9572** |
|  | **4.00** | **17.54630*** | **1.41200** | **.000** | **14.6539** | **20.4387** |
|  | **5.00** | **26.43519*** | **1.41200** | **.000** | **23.5428** | **29.3275** |
|  | **6.00** | **12.03704*** | **1.41200** | **.000** | **9.1447** | **14.9294** |
|  | **7.00** | **13.70370*** | **1.41200** | **.000** | **10.8113** | **16.5961** |
|  | **9.00** | **13.28704*** | **1.41200** | **.000** | **10.3947** | **16.1794** |
|  | **10.00** | **6.89815*** | **1.41200** | **.000** | **4.0058** | **9.7905** |
|  | **11.00** | **-1.75926** | **1.41200** | **.223** | **-4.6516** | **1.1331** |
|  | **12.00** | **-10.43981*** | **1.41200** | **.000** | **-13.3322** | **-7.5475** |
|  | **13.00** | **-7.31248*** | **1.41200** | **.000** | **-10.2048** | **-4.4201** |
|  | **14.00** | **-16.48915*** | **1.41200** | **.000** | **-19.3815** | **-13.5968** |
| **9.00** | **1.00** | **4.62963*** | **1.41200** | **.003** | **1.7373** | **7.5220** |
|  | **2.00** | **-3.70370*** | **1.41200** | **.014** | **-6.5961** | **-.8113** |
|  | **3.00** | **7.77778*** | **1.41200** | **.000** | **4.8854** | **10.6701** |
|  | **4.00** | **4.25926*** | **1.41200** | **.005** | **1.3669** | **7.1516** |
|  | **5.00** | **13.14815*** | **1.41200** | **.000** | **10.2558** | **16.0405** |
|  | **6.00** | **-1.25000** | **1.41200** | **.384** | **-4.1424** | **1.6424** |
|  | **7.00** | **.41667** | **1.41200** | **.770** | **-2.4757** | **3.3090** |
|  | **8.00** | **-13.28704*** | **1.41200** | **.000** | **-16.1794** | **-10.3947** |
|  | **10.00** | **-6.38889*** | **1.41200** | **.000** | **-9.2812** | **-3.4965** |
|  | **11.00** | **-15.04630*** | **1.41200** | **.000** | **-17.9387** | **-12.1539** |
|  | **12.00** | **-23.72685*** | **1.41200** | **.000** | **-26.6192** | **-20.8345** |
|  | **13.00** | **-20.59952*** | **1.41200** | **.000** | **-23.4919** | **-17.7072** |
|  | **14.00** | **-29.77619*** | **1.41200** | **.000** | **-32.6685** | **-26.8838** |
| **10.00** | **1.00** | **11.01852*** | **1.41200** | **.000** | **8.1262** | **13.9109** |
|  | **2.00** | **2.68519** | **1.41200** | **.068** | **-.2072** | **5.5775** |
|  | **3.00** | **14.16667*** | **1.41200** | **.000** | **11.2743** | **17.0590** |
|  | **4.00** | **10.64815*** | **1.41200** | **.000** | **7.7558** | **13.5405** |
|  | **5.00** | **19.53704*** | **1.41200** | **.000** | **16.6447** | **22.4294** |
|  | **6.00** | **5.13889*** | **1.41200** | **.001** | **2.2465** | **8.0312** |
|  | **7.00** | **6.80556*** | **1.41200** | **.000** | **3.9132** | **9.6979** |
|  | **8.00** | **-6.89815*** | **1.41200** | **.000** | **-9.7905** | **-4.0058** |
|  | **9.00** | **6.38889*** | **1.41200** | **.000** | **3.4965** | **9.2812** |
|  | **11.00** | **-8.65741*** | **1.41200** | **.000** | **-11.5498** | **-5.7651** |
|  | **12.00** | **-17.33796*** | **1.41200** | **.000** | **-20.2303** | **-14.4456** |
|  | **13.00** | **-14.21063*** | **1.41200** | **.000** | **-17.1030** | **-11.3183** |
|  | **14.00** | **-23.38730*** | **1.41200** | **.000** | **-26.2797** | **-20.4949** |
| **11.00** | **1.00** | **19.67593*** | **1.41200** | **.000** | **16.7836** | **22.5683** |
|  | **2.00** | **11.34259*** | **1.41200** | **.000** | **8.4502** | **14.2349** |
|  | **3.00** | **22.82407*** | **1.41200** | **.000** | **19.9317** | **25.7164** |
|  | **4.00** | **19.30556*** | **1.41200** | **.000** | **16.4132** | **22.1979** |
|  | **5.00** | **28.19444*** | **1.41200** | **.000** | **25.3021** | **31.0868** |
|  | **6.00** | **13.79630*** | **1.41200** | **.000** | **10.9039** | **16.6887** |
|  | **7.00** | **15.46296*** | **1.41200** | **.000** | **12.5706** | **18.3553** |
|  | **8.00** | **1.75926** | **1.41200** | **.223** | **-1.1331** | **4.6516** |
|  | **9.00** | **15.04630*** | **1.41200** | **.000** | **12.1539** | **17.9387** |
|  | **10.00** | **8.65741*** | **1.41200** | **.000** | **5.7651** | **11.5498** |
|  | **12.00** | **-8.68055*** | **1.41200** | **.000** | **-11.5729** | **-5.7882** |
|  | **13.00** | **-5.55322*** | **1.41200** | **.001** | **-8.4456** | **-2.6609** |
|  | **14.00** | **-14.72989*** | **1.41200** | **.000** | **-17.6222** | **-11.8375** |
| **12.00** | **1.00** | **28.35648*** | **1.41200** | **.000** | **25.4641** | **31.2488** |
|  | **2.00** | **20.02315*** | **1.41200** | **.000** | **17.1308** | **22.9155** |
|  | **3.00** | **31.50463*** | **1.41200** | **.000** | **28.6123** | **34.3970** |
|  | **4.00** | **27.98611*** | **1.41200** | **.000** | **25.0938** | **30.8785** |
|  | **5.00** | **36.87500*** | **1.41200** | **.000** | **33.9826** | **39.7674** |
|  | **6.00** | **22.47685*** | **1.41200** | **.000** | **19.5845** | **25.3692** |
|  | **7.00** | **24.14352*** | **1.41200** | **.000** | **21.2512** | **27.0359** |
|  | **8.00** | **10.43981*** | **1.41200** | **.000** | **7.5475** | **13.3322** |
|  | **9.00** | **23.72685*** | **1.41200** | **.000** | **20.8345** | **26.6192** |
|  | **10.00** | **17.33796*** | **1.41200** | **.000** | **14.4456** | **20.2303** |
|  | **11.00** | **8.68055*** | **1.41200** | **.000** | **5.7882** | **11.5729** |
|  | **13.00** | **3.12733*** | **1.41200** | **.035** | **.2350** | **6.0197** |
|  | **14.00** | **-6.04933*** | **1.41200** | **.000** | **-8.9417** | **-3.1570** |
| **13.00** | **1.00** | **25.22915*** | **1.41200** | **.000** | **22.3368** | **28.1215** |
|  | **2.00** | **16.89581*** | **1.41200** | **.000** | **14.0035** | **19.7882** |
|  | **3.00** | **28.37730*** | **1.41200** | **.000** | **25.4849** | **31.2697** |
|  | **4.00** | **24.85878*** | **1.41200** | **.000** | **21.9664** | **27.7511** |
|  | **5.00** | **33.74767*** | **1.41200** | **.000** | **30.8553** | **36.6400** |
|  | **6.00** | **19.34952*** | **1.41200** | **.000** | **16.4572** | **22.2419** |
|  | **7.00** | **21.01619*** | **1.41200** | **.000** | **18.1238** | **23.9085** |
|  | **8.00** | **7.31248*** | **1.41200** | **.000** | **4.4201** | **10.2048** |
|  | **9.00** | **20.59952*** | **1.41200** | **.000** | **17.7072** | **23.4919** |
|  | **10.00** | **14.21063*** | **1.41200** | **.000** | **11.3183** | **17.1030** |
|  | **11.00** | **5.55322*** | **1.41200** | **.001** | **2.6609** | **8.4456** |
|  | **12.00** | **-3.12733*** | **1.41200** | **.035** | **-6.0197** | **-.2350** |
|  | **14.00** | **-9.17667*** | **1.41200** | **.000** | **-12.0690** | **-6.2843** |
| **14.00** | **1.00** | **34.40581*** | **1.41200** | **.000** | **31.5135** | **37.2982** |
|  | **2.00** | **26.07248*** | **1.41200** | **.000** | **23.1801** | **28.9648** |
|  | **3.00** | **37.55396*** | **1.41200** | **.000** | **34.6616** | **40.4463** |
|  | **4.00** | **34.03544*** | **1.41200** | **.000** | **31.1431** | **36.9278** |
|  | **5.00** | **42.92433*** | **1.41200** | **.000** | **40.0320** | **45.8167** |
|  | **6.00** | **28.52619*** | **1.41200** | **.000** | **25.6338** | **31.4185** |
|  | **7.00** | **30.19285*** | **1.41200** | **.000** | **27.3005** | **33.0852** |
|  | **8.00** | **16.48915*** | **1.41200** | **.000** | **13.5968** | **19.3815** |
|  | **9.00** | **29.77619*** | **1.41200** | **.000** | **26.8838** | **32.6685** |
|  | **10.00** | **23.38730*** | **1.41200** | **.000** | **20.4949** | **26.2797** |
|  | **11.00** | **14.72989*** | **1.41200** | **.000** | **11.8375** | **17.6222** |
|  | **12.00** | **6.04933*** | **1.41200** | **.000** | **3.1570** | **8.9417** |
|  | **13.00** | **9.17667*** | **1.41200** | **.000** | **6.2843** | **12.0690** |
| ***. The mean difference is significant at the 0.05 level.** | | | | | | |

**H) Nutrient limitation –Lipid**

| **SPSS *posthoc* code** | **Exp. condition** | **Axenic/ Co-cultivated** |
| --- | --- | --- |
| **1** | **Sulphate Limitation** | **T** |
| **2** |  | **T-PB** |
| **3** | **Metal Limitation** | **T** |
| **4** |  | **T-PB** |
| **5** | **Nitrate Limitation** | **T** |
| **6** |  | **T-PB** |
| **7** | **Two stage- Salinity increase** | **T** |
| **8** |  | **T-PB** |
| **9** | **Two stage-pH decrease** | **T** |
| **10** |  | **T-PB** |
| **11** | **Two stage-Nitrate Limitation** | **T** |
| **12** |  | **T-PB** |
| **13** | **O3-Control (Nutrient Replete)** | **T** |
| **14** |  | **T-PB** |

**Lipid**

| **(I) VAR00002** | | **Mean Difference (I-J)** | **Std. Error** | **Sig.** | **95% Confidence Interval** | |
| --- | --- | --- | --- | --- | --- | --- |
|  |  |  |  |  | **Lower Bound** | **Upper Bound** |
| **1.00** | **2.00** | **-.31776** | **.65124** | **.629** | **-1.6518** | **1.0162** |
|  | **3.00** | **-12.82979^*^** | **.65124** | **.000** | **-14.1638** | **-11.4958** |
|  | **4.00** | **-8.00827^*^** | **.65124** | **.000** | **-9.3423** | **-6.6743** |
|  | **5.00** | **-26.13632^*^** | **.65124** | **.000** | **-27.4703** | **-24.8023** |
|  | **6.00** | **-25.40578^*^** | **.65124** | **.000** | **-26.7398** | **-24.0718** |
|  | **7.00** | **-15.70015^*^** | **.65124** | **.000** | **-17.0341** | **-14.3662** |
|  | **8.00** | **-11.70225^*^** | **.65124** | **.000** | **-13.0362** | **-10.3682** |
|  | **9.00** | **-15.44833^*^** | **.65124** | **.000** | **-16.7823** | **-14.1143** |
|  | **10.00** | **-11.86544^*^** | **.65124** | **.000** | **-13.1994** | **-10.5314** |
|  | **11.00** | **-22.41072^*^** | **.65124** | **.000** | **-23.7447** | **-21.0767** |
|  | **12.00** | **-24.03799^*^** | **.65124** | **.000** | **-25.3720** | **-22.7040** |
|  | **13.00** | **-15.11410^*^** | **.65124** | **.000** | **-16.4481** | **-13.7801** |
|  | **14.00** | **-13.76043^*^** | **.65124** | **.000** | **-15.0944** | **-12.4264** |
| **2.00** | **1.00** | **.31776** | **.65124** | **.629** | **-1.0162** | **1.6518** |
|  | **3.00** | **-12.51203^*^** | **.65124** | **.000** | **-13.8460** | **-11.1780** |
|  | **4.00** | **-7.69051^*^** | **.65124** | **.000** | **-9.0245** | **-6.3565** |
|  | **5.00** | **-25.81856^*^** | **.65124** | **.000** | **-27.1526** | **-24.4846** |
|  | **6.00** | **-25.08802^*^** | **.65124** | **.000** | **-26.4220** | **-23.7540** |
|  | **7.00** | **-15.38239^*^** | **.65124** | **.000** | **-16.7164** | **-14.0484** |
|  | **8.00** | **-11.38449^*^** | **.65124** | **.000** | **-12.7185** | **-10.0505** |
|  | **9.00** | **-15.13057^*^** | **.65124** | **.000** | **-16.4646** | **-13.7966** |
|  | **10.00** | **-11.54768^*^** | **.65124** | **.000** | **-12.8817** | **-10.2137** |
|  | **11.00** | **-22.09296^*^** | **.65124** | **.000** | **-23.4270** | **-20.7590** |
|  | **12.00** | **-23.72023^*^** | **.65124** | **.000** | **-25.0542** | **-22.3862** |
|  | **13.00** | **-14.79634^*^** | **.65124** | **.000** | **-16.1303** | **-13.4623** |
|  | **14.00** | **-13.44267^*^** | **.65124** | **.000** | **-14.7767** | **-12.1087** |
| **3.00** | **1.00** | **12.82979^*^** | **.65124** | **.000** | **11.4958** | **14.1638** |
|  | **2.00** | **12.51203^*^** | **.65124** | **.000** | **11.1780** | **13.8460** |
|  | **4.00** | **4.82152^*^** | **.65124** | **.000** | **3.4875** | **6.1555** |
|  | **5.00** | **-13.30653^*^** | **.65124** | **.000** | **-14.6405** | **-11.9725** |
|  | **6.00** | **-12.57599^*^** | **.65124** | **.000** | **-13.9100** | **-11.2420** |
|  | **7.00** | **-2.87036^*^** | **.65124** | **.000** | **-4.2044** | **-1.5364** |
|  | **8.00** | **1.12754** | **.65124** | **.094** | **-.2065** | **2.4615** |
|  | **9.00** | **-2.61854^*^** | **.65124** | **.000** | **-3.9525** | **-1.2845** |
|  | **10.00** | **.96435** | **.65124** | **.150** | **-.3696** | **2.2984** |
|  | **11.00** | **-9.58093^*^** | **.65124** | **.000** | **-10.9149** | **-8.2469** |
|  | **12.00** | **-11.20820^*^** | **.65124** | **.000** | **-12.5422** | **-9.8742** |
|  | **13.00** | **-2.28431^*^** | **.65124** | **.002** | **-3.6183** | **-.9503** |
|  | **14.00** | **-.93064** | **.65124** | **.164** | **-2.2646** | **.4034** |
| **4.00** | **1.00** | **8.00827^*^** | **.65124** | **.000** | **6.6743** | **9.3423** |
|  | **2.00** | **7.69051^*^** | **.65124** | **.000** | **6.3565** | **9.0245** |
|  | **3.00** | **-4.82152^*^** | **.65124** | **.000** | **-6.1555** | **-3.4875** |
|  | **5.00** | **-18.12805^*^** | **.65124** | **.000** | **-19.4620** | **-16.7940** |
|  | **6.00** | **-17.39750^*^** | **.65124** | **.000** | **-18.7315** | **-16.0635** |
|  | **7.00** | **-7.69188^*^** | **.65124** | **.000** | **-9.0259** | **-6.3579** |
|  | **8.00** | **-3.69397^*^** | **.65124** | **.000** | **-5.0280** | **-2.3600** |
|  | **9.00** | **-7.44006^*^** | **.65124** | **.000** | **-8.7741** | **-6.1061** |
|  | **10.00** | **-3.85716^*^** | **.65124** | **.000** | **-5.1912** | **-2.5232** |
|  | **11.00** | **-14.40245^*^** | **.65124** | **.000** | **-15.7364** | **-13.0685** |
|  | **12.00** | **-16.02972^*^** | **.65124** | **.000** | **-17.3637** | **-14.6957** |
|  | **13.00** | **-7.10582^*^** | **.65124** | **.000** | **-8.4398** | **-5.7718** |
|  | **14.00** | **-5.75216^*^** | **.65124** | **.000** | **-7.0862** | **-4.4182** |
| **5.00** | **1.00** | **26.13632^*^** | **.65124** | **.000** | **24.8023** | **27.4703** |
|  | **2.00** | **25.81856^*^** | **.65124** | **.000** | **24.4846** | **27.1526** |
|  | **3.00** | **13.30653^*^** | **.65124** | **.000** | **11.9725** | **14.6405** |
|  | **4.00** | **18.12805^*^** | **.65124** | **.000** | **16.7940** | **19.4620** |
|  | **6.00** | **.73054** | **.65124** | **.271** | **-.6035** | **2.0645** |
|  | **7.00** | **10.43617^*^** | **.65124** | **.000** | **9.1022** | **11.7702** |
|  | **8.00** | **14.43407^*^** | **.65124** | **.000** | **13.1001** | **15.7681** |
|  | **9.00** | **10.68799^*^** | **.65124** | **.000** | **9.3540** | **12.0220** |
|  | **10.00** | **14.27088^*^** | **.65124** | **.000** | **12.9369** | **15.6049** |
|  | **11.00** | **3.72560^*^** | **.65124** | **.000** | **2.3916** | **5.0596** |
|  | **12.00** | **2.09833^*^** | **.65124** | **.003** | **.7643** | **3.4323** |
|  | **13.00** | **11.02222^*^** | **.65124** | **.000** | **9.6882** | **12.3562** |
|  | **14.00** | **12.37589^*^** | **.65124** | **.000** | **11.0419** | **13.7099** |
| **6.00** | **1.00** | **25.40578^*^** | **.65124** | **.000** | **24.0718** | **26.7398** |
|  | **2.00** | **25.08802^*^** | **.65124** | **.000** | **23.7540** | **26.4220** |
|  | **3.00** | **12.57599^*^** | **.65124** | **.000** | **11.2420** | **13.9100** |
|  | **4.00** | **17.39750^*^** | **.65124** | **.000** | **16.0635** | **18.7315** |
|  | **5.00** | **-.73054** | **.65124** | **.271** | **-2.0645** | **.6035** |
|  | **7.00** | **9.70563^*^** | **.65124** | **.000** | **8.3716** | **11.0396** |
|  | **8.00** | **13.70353^*^** | **.65124** | **.000** | **12.3695** | **15.0375** |
|  | **9.00** | **9.95745^*^** | **.65124** | **.000** | **8.6234** | **11.2914** |
|  | **10.00** | **13.54034^*^** | **.65124** | **.000** | **12.2063** | **14.8743** |
|  | **11.00** | **2.99505^*^** | **.65124** | **.000** | **1.6611** | **4.3291** |
|  | **12.00** | **1.36779^*^** | **.65124** | **.045** | **.0338** | **2.7018** |
|  | **13.00** | **10.29168^*^** | **.65124** | **.000** | **8.9577** | **11.6257** |
|  | **14.00** | **11.64534^*^** | **.65124** | **.000** | **10.3113** | **12.9793** |
| **7.00** | **1.00** | **15.70015^*^** | **.65124** | **.000** | **14.3662** | **17.0341** |
|  | **2.00** | **15.38239^*^** | **.65124** | **.000** | **14.0484** | **16.7164** |
|  | **3.00** | **2.87036^*^** | **.65124** | **.000** | **1.5364** | **4.2044** |
|  | **4.00** | **7.69188^*^** | **.65124** | **.000** | **6.3579** | **9.0259** |
|  | **5.00** | **-10.43617^*^** | **.65124** | **.000** | **-11.7702** | **-9.1022** |
|  | **6.00** | **-9.70563^*^** | **.65124** | **.000** | **-11.0396** | **-8.3716** |
|  | **8.00** | **3.99790^*^** | **.65124** | **.000** | **2.6639** | **5.3319** |
|  | **9.00** | **.25182** | **.65124** | **.702** | **-1.0822** | **1.5858** |
|  | **10.00** | **3.83472^*^** | **.65124** | **.000** | **2.5007** | **5.1687** |
|  | **11.00** | **-6.71057^*^** | **.65124** | **.000** | **-8.0446** | **-5.3766** |
|  | **12.00** | **-8.33784^*^** | **.65124** | **.000** | **-9.6718** | **-7.0038** |
|  | **13.00** | **.58605** | **.65124** | **.376** | **-.7479** | **1.9201** |
|  | **14.00** | **1.93972^*^** | **.65124** | **.006** | **.6057** | **3.2737** |
| **8.00** | **1.00** | **11.70225^*^** | **.65124** | **.000** | **10.3682** | **13.0362** |
|  | **2.00** | **11.38449^*^** | **.65124** | **.000** | **10.0505** | **12.7185** |
|  | **3.00** | **-1.12754** | **.65124** | **.094** | **-2.4615** | **.2065** |
|  | **4.00** | **3.69397^*^** | **.65124** | **.000** | **2.3600** | **5.0280** |
|  | **5.00** | **-14.43407^*^** | **.65124** | **.000** | **-15.7681** | **-13.1001** |
|  | **6.00** | **-13.70353^*^** | **.65124** | **.000** | **-15.0375** | **-12.3695** |
|  | **7.00** | **-3.99790^*^** | **.65124** | **.000** | **-5.3319** | **-2.6639** |
|  | **9.00** | **-3.74608^*^** | **.65124** | **.000** | **-5.0801** | **-2.4121** |
|  | **10.00** | **-.16319** | **.65124** | **.804** | **-1.4972** | **1.1708** |
|  | **11.00** | **-10.70848^*^** | **.65124** | **.000** | **-12.0425** | **-9.3745** |
|  | **12.00** | **-12.33574^*^** | **.65124** | **.000** | **-13.6697** | **-11.0017** |
|  | **13.00** | **-3.41185^*^** | **.65124** | **.000** | **-4.7459** | **-2.0779** |
|  | **14.00** | **-2.05818^*^** | **.65124** | **.004** | **-3.3922** | **-.7242** |
| **9.00** | **1.00** | **15.44833^*^** | **.65124** | **.000** | **14.1143** | **16.7823** |
|  | **2.00** | **15.13057^*^** | **.65124** | **.000** | **13.7966** | **16.4646** |
|  | **3.00** | **2.61854^*^** | **.65124** | **.000** | **1.2845** | **3.9525** |
|  | **4.00** | **7.44006^*^** | **.65124** | **.000** | **6.1061** | **8.7741** |
|  | **5.00** | **-10.68799^*^** | **.65124** | **.000** | **-12.0220** | **-9.3540** |
|  | **6.00** | **-9.95745^*^** | **.65124** | **.000** | **-11.2914** | **-8.6234** |
|  | **7.00** | **-.25182** | **.65124** | **.702** | **-1.5858** | **1.0822** |
|  | **8.00** | **3.74608^*^** | **.65124** | **.000** | **2.4121** | **5.0801** |
|  | **10.00** | **3.58289^*^** | **.65124** | **.000** | **2.2489** | **4.9169** |
|  | **11.00** | **-6.96239^*^** | **.65124** | **.000** | **-8.2964** | **-5.6284** |
|  | **12.00** | **-8.58966^*^** | **.65124** | **.000** | **-9.9237** | **-7.2557** |
|  | **13.00** | **.33423** | **.65124** | **.612** | **-.9998** | **1.6682** |
|  | **14.00** | **1.68790^*^** | **.65124** | **.015** | **.3539** | **3.0219** |
| **10.00** | **1.00** | **11.86544^*^** | **.65124** | **.000** | **10.5314** | **13.1994** |
|  | **2.00** | **11.54768^*^** | **.65124** | **.000** | **10.2137** | **12.8817** |
|  | **3.00** | **-.96435** | **.65124** | **.150** | **-2.2984** | **.3696** |
|  | **4.00** | **3.85716^*^** | **.65124** | **.000** | **2.5232** | **5.1912** |
|  | **5.00** | **-14.27088^*^** | **.65124** | **.000** | **-15.6049** | **-12.9369** |
|  | **6.00** | **-13.54034^*^** | **.65124** | **.000** | **-14.8743** | **-12.2063** |
|  | **7.00** | **-3.83472^*^** | **.65124** | **.000** | **-5.1687** | **-2.5007** |
|  | **8.00** | **.16319** | **.65124** | **.804** | **-1.1708** | **1.4972** |
|  | **9.00** | **-3.58289^*^** | **.65124** | **.000** | **-4.9169** | **-2.2489** |
|  | **11.00** | **-10.54529^*^** | **.65124** | **.000** | **-11.8793** | **-9.2113** |
|  | **12.00** | **-12.17255^*^** | **.65124** | **.000** | **-13.5066** | **-10.8386** |
|  | **13.00** | **-3.24866^*^** | **.65124** | **.000** | **-4.5827** | **-1.9147** |
|  | **14.00** | **-1.89500^*^** | **.65124** | **.007** | **-3.2290** | **-.5610** |
| **11.00** | **1.00** | **22.41072^*^** | **.65124** | **.000** | **21.0767** | **23.7447** |
|  | **2.00** | **22.09296^*^** | **.65124** | **.000** | **20.7590** | **23.4270** |
|  | **3.00** | **9.58093^*^** | **.65124** | **.000** | **8.2469** | **10.9149** |
|  | **4.00** | **14.40245^*^** | **.65124** | **.000** | **13.0685** | **15.7364** |
|  | **5.00** | **-3.72560^*^** | **.65124** | **.000** | **-5.0596** | **-2.3916** |
|  | **6.00** | **-2.99505^*^** | **.65124** | **.000** | **-4.3291** | **-1.6611** |
|  | **7.00** | **6.71057^*^** | **.65124** | **.000** | **5.3766** | **8.0446** |
|  | **8.00** | **10.70848^*^** | **.65124** | **.000** | **9.3745** | **12.0425** |
|  | **9.00** | **6.96239^*^** | **.65124** | **.000** | **5.6284** | **8.2964** |
|  | **10.00** | **10.54529^*^** | **.65124** | **.000** | **9.2113** | **11.8793** |
|  | **12.00** | **-1.62727^*^** | **.65124** | **.019** | **-2.9613** | **-.2933** |
|  | **13.00** | **7.29662^*^** | **.65124** | **.000** | **5.9626** | **8.6306** |
|  | **14.00** | **8.65029^*^** | **.65124** | **.000** | **7.3163** | **9.9843** |
| **12.00** | **1.00** | **24.03799^*^** | **.65124** | **.000** | **22.7040** | **25.3720** |
|  | **2.00** | **23.72023^*^** | **.65124** | **.000** | **22.3862** | **25.0542** |
|  | **3.00** | **11.20820^*^** | **.65124** | **.000** | **9.8742** | **12.5422** |
|  | **4.00** | **16.02972^*^** | **.65124** | **.000** | **14.6957** | **17.3637** |
|  | **5.00** | **-2.09833^*^** | **.65124** | **.003** | **-3.4323** | **-.7643** |
|  | **6.00** | **-1.36779^*^** | **.65124** | **.045** | **-2.7018** | **-.0338** |
|  | **7.00** | **8.33784^*^** | **.65124** | **.000** | **7.0038** | **9.6718** |
|  | **8.00** | **12.33574^*^** | **.65124** | **.000** | **11.0017** | **13.6697** |
|  | **9.00** | **8.58966^*^** | **.65124** | **.000** | **7.2557** | **9.9237** |
|  | **10.00** | **12.17255^*^** | **.65124** | **.000** | **10.8386** | **13.5066** |
|  | **11.00** | **1.62727^*^** | **.65124** | **.019** | **.2933** | **2.9613** |
|  | **13.00** | **8.92389^*^** | **.65124** | **.000** | **7.5899** | **10.2579** |
|  | **14.00** | **10.27756^*^** | **.65124** | **.000** | **8.9436** | **11.6116** |
| **13.00** | **1.00** | **15.11410^*^** | **.65124** | **.000** | **13.7801** | **16.4481** |
|  | **2.00** | **14.79634^*^** | **.65124** | **.000** | **13.4623** | **16.1303** |
|  | **3.00** | **2.28431^*^** | **.65124** | **.002** | **.9503** | **3.6183** |
|  | **4.00** | **7.10582^*^** | **.65124** | **.000** | **5.7718** | **8.4398** |
|  | **5.00** | **-11.02222^*^** | **.65124** | **.000** | **-12.3562** | **-9.6882** |
|  | **6.00** | **-10.29168^*^** | **.65124** | **.000** | **-11.6257** | **-8.9577** |
|  | **7.00** | **-.58605** | **.65124** | **.376** | **-1.9201** | **.7479** |
|  | **8.00** | **3.41185^*^** | **.65124** | **.000** | **2.0779** | **4.7459** |
|  | **9.00** | **-.33423** | **.65124** | **.612** | **-1.6682** | **.9998** |
|  | **10.00** | **3.24866^*^** | **.65124** | **.000** | **1.9147** | **4.5827** |
|  | **11.00** | **-7.29662^*^** | **.65124** | **.000** | **-8.6306** | **-5.9626** |
|  | **12.00** | **-8.92389^*^** | **.65124** | **.000** | **-10.2579** | **-7.5899** |
|  | **14.00** | **1.35367^*^** | **.65124** | **.047** | **.0197** | **2.6877** |
| **14.00** | **1.00** | **13.76043^*^** | **.65124** | **.000** | **12.4264** | **15.0944** |
|  | **2.00** | **13.44267^*^** | **.65124** | **.000** | **12.1087** | **14.7767** |
|  | **3.00** | **.93064** | **.65124** | **.164** | **-.4034** | **2.2646** |
|  | **4.00** | **5.75216^*^** | **.65124** | **.000** | **4.4182** | **7.0862** |
|  | **5.00** | **-12.37589^*^** | **.65124** | **.000** | **-13.7099** | **-11.0419** |
|  | **6.00** | **-11.64534^*^** | **.65124** | **.000** | **-12.9793** | **-10.3113** |
|  | **7.00** | **-1.93972^*^** | **.65124** | **.006** | **-3.2737** | **-.6057** |
|  | **8.00** | **2.05818^*^** | **.65124** | **.004** | **.7242** | **3.3922** |
|  | **9.00** | **-1.68790^*^** | **.65124** | **.015** | **-3.0219** | **-.3539** |
|  | **10.00** | **1.89500^*^** | **.65124** | **.007** | **.5610** | **3.2290** |
|  | **11.00** | **-8.65029^*^** | **.65124** | **.000** | **-9.9843** | **-7.3163** |
|  | **12.00** | **-10.27756^*^** | **.65124** | **.000** | **-11.6116** | **-8.9436** |
|  | **13.00** | **-1.35367^*^** | **.65124** | **.047** | **-2.6877** | **-.0197** |
| ***. The mean difference is significant at the 0.05 level.** | | | | | | |
